# Co-crystals, Salts or Mixtures of Both? The Case of Tenofovir Alafenamide Fumarates

**DOI:** 10.3390/pharmaceutics12040342

**Published:** 2020-04-10

**Authors:** Hannes Lengauer, Damjan Makuc, Damjan Šterk, Franc Perdih, Arthur Pichler, Tina Trdan Lušin, Janez Plavec, Zdenko Časar

**Affiliations:** 1 Sandoz GmbH, Early Stage Development, API Characterization, Biochemiestraße 10, AT-6250 Kundl, Austria; hannes.lengauer@sandoz.com (H.L.); arthur.pichler@sandoz.com (A.P.); 2 National Institute of Chemistry, Slovenian NMR Centre, Hajdrihova 19, SI-1001 Ljubljana, Slovenia; damjan.makuc@ki.si (D.M.); janez.plavec@ki.si (J.P.); 3 EN-FIST Centre of Excellence, Trg osvobodilne fronte 13, SI-1000 Ljubljana, Slovenia; 4 Lek Pharmaceuticals d.d., Sandoz Development Center Slovenia, Verovškova ulica 57, SI-1526 Ljubljana, Slovenia; damjan.sterk@sandoz.com (D.Š.); tina.trdan_lusin@sandoz.com (T.T.L.); 5 University of Ljubljana, Faculty of Chemistry and Chemical Technology, Večna pot 113, SI-1001 Ljubljana, Slovenia; franc.perdih@fkkt.uni-lj.si; 6 Faculty of Pharmacy, University of Ljubljana, Aškerčeva cesta 7, SI-1000 Ljubljana, Slovenia

**Keywords:** co-crystal, salt, polymorphism, ssNMR, X-ray diffraction, tenofovir alafenamide fumarate

## Abstract

Tenofovir alafenamide fumarate (TAF) is the newest prodrug of tenofovir that constitutes several drug products used for the treatment of HIV/AIDS. Although the solid-state properties of its predecessor tenofovir disoproxil fumarate have been investigated and described in the literature, there are no data in the scientific literature on the solid state properties of TAF. In our report, we describe the preparation of two novel polymorphs II and III of tenofovir alafenamide monofumarate (TA MF2 and TA MF3). The solid-state structure of these compounds was investigated in parallel to the previously known tenofovir alafenamide monofumarate form I (TA MF1) and tenofovir alafenamide hemifumarate (TA HF). Interestingly, the single-crystal X-ray diffraction of TA HF revealed that this derivative exists as a co-crystal form. In addition, we prepared a crystalline tenofovir alafenamide free base (TA) and its hydrochloride salt (TA HCl), which enabled us to determine the structure of TA MF derivatives using ^15^N-ssNMR (^15^N-solid state nuclear magnetic resonance). Surprisingly, we observed that TA MF1 exists as a mixed ionization state complex or pure salt, while TA MF2 and TA MF3 can be obtained as pure co-crystal forms.

## 1. Introduction

HIV/AIDS remains one of the important causes of death globally and the leading cause of death in low-income countries [1]. It was estimated that nearly 38 million people were living with HIV in 2018 and that 770,000 died because of AIDS globally in 2018 [2]. There are several therapeutic classes of drugs available for the treatment of HIV infection [3,4,5]. Tenofovir alafenamide fumarate (known as TAF) [6,7,8] is a novel prodrug derivative of the well-known nucleotide reverse transcriptase inhibitor (NtRTI) tenofovir (TFV) [9,10,11], which was initially developed for the treatment of HIV infection as tenofovir disoproxil fumarate (TDF) in the late 1990s [12,13,14] and marketed under the tradename Viread^®^ since 2001 [15]. TAF, as a new type of TFV prodrug, was approved as a mono therapy (Vemlidy^®^) in November 2016 for the treatment of chronic hepatitis B virus infection [16,17,18,19,20] in adults with compensated liver disease by the U.S Food & Drug Administration (FDA). Interestingly, according to the FDA data, TAF is marketed as a hemifumarate [21]. In addition, TAF received approval by the FDA for the treatment of HIV infection as a fixed dose combination with: cobicistat, elvitegravir and emtricitabine (Genvoya^®^) in November 2015 [22,23,24]; emtricitabine and rilpivirine hydrochloride (Odefsey^®^) in March 2016 [25]; emtricitabine (Descovy^®^) in April 2016 [26]; bictegravir sodium and emtricitabine (Biktarvy^®^) in February 2018; and cobicistat, darunavir and emtricitabine (Symtuza^®^) in July 2018 [27,28,29]. TAF exhibited an improved antiviral activity and safety profile related to renal and bone toxicity [30,31,32]. Therefore, TFV, TDF and in particular TAF represent key compounds for the treatment of chronic hepatitis B and HIV infections and are thus a subject of intense research in the area of new drug delivery systems [33,34,35,36,37,38,39,40].

Polymorphism plays a pivotal role in drug performance, because it affects a number of drug properties like chemical and physical stability, hygroscopicity, solubility, dissolution rate, flowability, compressibility, bioavailability and efficacy [41,42,43,44,45,46,47,48,49,50,51,52,53,54,55,56,57,58,59]. Moreover, in recent years, pharmaceutical co-crystals have emerged as a promising option to alter drug properties by tailoring their physicochemical and biopharmaceutical attributes [60,61,62,63,64,65,66,67,68,69,70,71,72,73,74,75,76,77,78,79,80,81,82,83,84,85,86,87,88,89,90,91,92]. Surprisingly, until now only three drugs were registered and marketed as pharmaceutical co-crystals [93]. 

**Figure 1:**
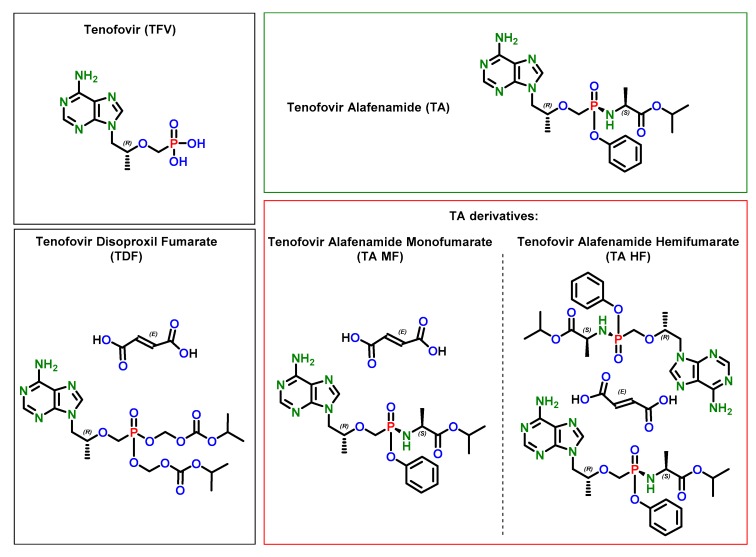
Chemical structure of tenofovir and its prodrug fumarate derivatives.

Interestingly, although there are several literature reports on the solid-state properties of TDF [94,95,96,97,98,99], to the best of our knowledge, TAF solid-state chemistry has not yet been described in the scientific literature. The patent literature discloses tenofovir alafenamide monofumarate form I (TA MF1) [100], as well as tenofovir alafenamide hemifumarate (TA HF) [101], which is considered to be the more thermodynamically stable form. Recently, we disclosed the preparation of two novel tenofovir alafenamide monofumarate forms II and III: TA MF2 and TA MF3 [102] (Figure 1).

Due to the molecular structure of tenofovir alafenamide, which contains an adenine heterocyclic core with several nitrogen atoms (p*K*_a1_ = 4.2, p*K*_a2_ = 9.8) [103,104,105,106] and the acidity of fumaric acid (p*K*_a1_ = 3.0, p*K*_a2_ = 4.4) [107], this acid–base pair has a small Δp*K*_a_ (p*K*_a_ of base - p*K*_a_ of acid) value of ca. 1.2. Based on the known Δp*K*_a_ rule [64], the continuum between the salt and co-crystal states can be expected in such a case. Since the distinction between salts and co-crystals has important regulatory implications [108], we were prompted to investigate whether the abovementioned tenofovir alafenamide derivatives exist as salts, co-crystals or complexes with mixed ionized states. Moreover, we were interested in determining potential protonation sites in tenofovir alafenamide. In the case of TA, which contains six nitrogen atoms, ^15^N-ssNMR (^15^N-solid state nuclear magnetic resonance) provides an interesting option for the elucidation of the ionization state of TA HF and TA MFs. The magic angle spinning (MAS) nuclear magnetic resonance (NMR) structure characterization of tenofovir alafenamide analogues was corroborated by the Lee-Goldburg cross-polarization method (LG-CP) [109,110]. It was shown that ^1^H-^13^C distances can be obtained with high precision from LG-CP with fast MAS and continuous LG decoupling on uniformly ^13^C-enriched tyrosine hydrochloride [111]. The Lee-Goldburg cross-polarization pulse sequence offers an interesting choice for obtaining a useful spectral editing opportunity. This strategy has been applied to identify the nitrogen atoms directly bonded to protons using ^15^N MAS NMR spectroscopy. Its Fourier transform yields an effective ^15^N frequency response that is very sensitive to the surrounding protons. For the protons directly bonded to a ^15^N, the magnetization is transferred in a short time (200 μs was used to transfer the polarization in our case), whereas no signal would be observed for non-protonated nitrogen atoms, which offers an attractive method for the characterization of intermolecular hydrogen bonding and protonated species. 

Surprisingly, in our study a combination of ^15^N-ssNMR and single-crystal X-ray diffraction, revealed that TA HF exists as a co-crystal, TA MF1 as a mixture of the mixed ionization state or pure salt, while TA MF2 and TA MF3 can be obtained as pure co-crystal structures.

## 2. Materials and Methods 

### 2.1. Materials

For the purpose of this study, the following materials were used: TA HF (Laurus Labs, Hyderabad, India), TA MF1 (Honour Lab Limited, Hyderabad, India; Cipla, Mumbai, India; Lek, Mengeš, Slovenia), TA MF2 (Sandoz GmbH, Kundl, Austria) and TA MF3 (Sandoz GmbH, Kundl, Austria).

For the purpose of the tenofovir alafenamide and tenofovir alafenamide hydrochloride synthesis, tenofovir alafenamide hemifumarate was purchased from Jiangsu Cdymax Pharmaceuticals Co., Ltd. (Qidong, China). Sodium hydrogen carbonate, hydrochloric acid, sulfuric acid, acetonitrile and *tert*-butyl methyl ether were purchased from Merck (Darmstadt, Germany). Sodium sulfate and dichloromethane were purchased from Sigma-Aldrich (St. Louis, MO, USA).

### 2.2. Characterization Methods

#### 2.2.1. Attenuated Total Reflection Fourier-Transform Infrared (ATR-FTIR) Measurements

ATR-FTIR spectra were collected with FTIR spectrometer Spectrum Two (PerkinElmer, Waltham, MA, USA), using a single reflection diamond ATR cell.

#### 2.2.2. Solution Nuclear Magnetic Resonance (NMR) Spectroscopy

All solution NMR spectra were recorded at 298 K on a Varian VNMR400 NMR spectrometer (Varian Inc., Palo Alto, CA, USA) equipped with an AutoX DB double resonance probe operating at a **^1^**H resonance frequency of 400 MHz and a **^13^**C resonance frequency of 100 MHz. **^1^**H NMR chemical shifts (*δ_H_*) and **^13^**C NMR chemical shifts (*δ_C_*) are quoted in parts per million (ppm) downfield from tetramethylsilane (TMS), and coupling constants (*J*) are quoted in Hertz (Hz). Abbreviations for NMR data are s (singlet), d (doublet), t (triplet), sept (septet) and m (multiplet).

#### 2.2.3. Solid-State Nuclear Magnetic Resonance Analysis

NMR spectra were acquired on the Agilent Technologies NMR System 600 MHz NMR spectrometer (Varian Inc., Palo Alto, CA, USA) equipped with a 3.2 mm NB dual resonance HX MAS probe. The Larmor frequencies of proton and nitrogen nuclei were 599.52 and 60.77 MHz, respectively. ^1^H NMR chemical shifts are reported relative to external reference adamantane (*δ_H_* 1.85 ppm), which corresponds to the TMS signal at *δ_H_* 0.0 ppm. ^15^N-NMR chemical shifts are reported relative to ammonium sulfate (*δ_N_* −355.7 ppm), which corresponds to the nitromethane signal at *δ_N_* 0.0 ppm. Samples were spun at 16,000 (^1^H) and 10,000 Hz (^15^N). A short excitation time of 200 μs was used to transfer polarization, a relaxation delay of 1 s and at least 200,000 repetitions.

#### 2.2.4. DSC Measurements

DSC thermograms were acquired using the differential scanning calorimeter DSC 1 instrument (Mettler Toledo, Polaris Parkway Columbus, OH, USA) operating at 10 °C/min.

#### 2.2.5. p-XRD Measurements

Powder X-ray diffraction patterns (*p*-XRD) of newly prepared (TA MF2 and TA MF3) or sourced (TA HF and TA MF1) tenofovir alafenamide fumarate derivatives were obtained with an X’Pert PRO diffractometer (PANalytical, Almelo, Netherlands) equipped with a theta/theta coupled goniometer in transmission geometry, using a programmable XYZ-stage with a well plate holder, a Cu-Kα radiation source (wavelength 0,15419 nm) with a focusing mirror, a 0.5° divergence slit, a 0.04 rad Soller slit collimator and a 0.5° anti-scattering slit on the incident beam side, a 1.4 mm anti-scattering slit, a 0.02 rad Soller slit collimator, a Ni-filter and a 1d-PIXcel solid state line detector (255 channels) on the diffracted beam side. Diffractograms were recorded at a tube voltage of 45 kV, tube current of 40 mA, applying a stepsize of 0.013° 2θ with an exposure time of 40 s per step in the angular range of 2° to 40° 2θ under ambient conditions. For the displayed figure, diffractograms were first adapted to uniform maximum peak intensities and then shifted vertically. Since no characteristic reflections were visible above 30° 2θ, the diffractograms are shown in the range of 2–30° 2θ.

Bulk powder samples of TA and TA HCl were analyzed by powder X-ray diffraction using the PANalytical Empyrean diffractometer (Malvern Panalytical Almelo, Netherland). Powder samples were analyzed under the following instrumental conditions: CuKα radiation (45 kV, 40 mA); scan range 2–40° 2θ; step 0.026° 2θ; and time per step 50 s. Automatic divergence and antiscatter slits were used to irradiate 10 mm of sample length.

#### 2.2.6. X-Ray Single Crystal Analysis

Single-crystal X-ray diffraction data of TA and TA HF were collected on an Agilent Technologies SuperNova Dual diffractometer *(*Yarnton, UK*)* with an Atlas detector using monochromated Cu-*K*α radiation (*λ* = 1.54184 Å) at room temperature (TA) or 150 K (TA HF). The data were processed using CrysAlis Pro [112]. The structures were solved by the Superflip program [113] using charge-flipping methods and were refined by a full-matrix least-squares procedure based on *F^2^* with SHELX2014 [114] using the Olex2 program suite [115]. All non-hydrogen atoms were refined anisotropically. All hydrogen atoms were readily located in difference Fourier maps. Hydrogen atoms bonded to carbon atoms were subsequently treated as riding atoms in geometrically idealized positions with *U*_iso_(H) = *kU*_eq_(C), where *k* = 1.5 for methyl groups, which were permitted to rotate but not to tilt, and 1.2 for all other H atoms. Hydrogen atoms bonded to nitrogen atoms were refined fixing the bond lengths and isotropic temperature factors as *U*_iso_(H) = 1.2*U*_eq_(N). In TA HF, the hydrogen atom bonded to the O6 atom of fumaric acid was readily located from difference Fourier maps and was treated by fixing the coordinates and isotropic temperature factors as *U*_iso_(H) = 1.5*U*_eq_(O). Isopropyl and phenyl groups in TA HF were found disordered over two positions with a refined ratio of 0.773(10):0.227(10) and 0.554(3):0.446(3), respectively. The crystallographic data are listed in Table 1.

### 2.3. Synthesis and Characterization of Tenofovir Alafenamide Derivatives

#### 2.3.1. Synthesis of Tenofovir Alafenamide (TA)

Dichloromethane (30 mL) was added to a mixture of tenofovir alafenamide hemifumarate (TA HF) (5.00 g, 9.35 mmol of tenofovir alafenamide), sodium hydrogen carbonate (0.85 g, 10.1 mmol) and water (10 mL). The phases were separated, and the water phase was washed with dichloromethane (10 mL). Combined dichloromethane phases were dried over sodium sulfate, and then the solids were filtered off. The filtrate was concentrated to half of the initial volume on the rotary evaporator, *tert*-butyl methyl ether (75 mL) was added to the solution, and the mixture was again concentrated on the rotary evaporator. *Tert*-butyl methyl ether (20 mL) was added to the mixture, the solids were filtered off, washed with *tert*-butyl methyl ether (20 mL) and dried at 40 °C to obtain tenofovir alafenamide as white powder. Yield: 4.34 g (97% of theory). Suitable crystals for the single crystal X-ray analysis were obtained by heating a mixture of tenofovir alafenamide (200 mg) in acetonitrile (5 mL) until complete dissolution, subsequent slow cooling to ~4 °C and isolation by filtration. DSC (10 °K/min): 122.28 °C onset, 123.92 °C peak (literature data [116]: mp 117–120 °C); ATR-FTIR: 466, 481, 540, 554, 571, 617, 666, 694, 725, 776, 799, 839, 890, 903, 920, 997, 1009, 1068, 1092, 1115, 1133, 1162, 1199, 1226, 1248, 1271, 1303, 1326, 1349, 1374, 1417, 1455, 1487, 1575, 1600, 1674, 1722, 2940, 2979, 3133 cm^−1^; ^1^H-NMR (DMSO-*d_6_*, 400 MHz): *δ* 1.07 (d, 3H, *J* = 6.2 Hz), 1.11–1.06 (m, 9H), 3.73 (dd, 1H, *J* = 13.2, 9.7 Hz), 3.81-3.94 (m, 3H), 4.13 (dd, 1H, *J* = 14.4, 6.6 Hz), 4.26 (dd, 1H, *J* = 14.4, 3.9 Hz), 4.84 (sept, 1H, *J* = 6.2 Hz), 5.60 (dd, 1H, *J* = 11.7, 10.5 Hz), 7.02 (m, 2H), 7.09 (m, 1H), 7.14 (s, 2H), 7.25 (m, 2H), 8.07 (s, 1H), 8.11 (s, 1H) ppm; ^13^C-NMR (DMSO-*d_6_*, 100 MHz, proton decoupled): *δ* 16.7, 20.5, 21.5, 21.6, 47.0, 49.2, 64.3 (d, *J* = 156 Hz), 68.0, 75.7 (d, *J* = 13 Hz), 118.6, 120.6 (d, *J* = 5 Hz), 124.4, 129.5, 141.4, 149.9, 150.4, 152.5, 156.1, 173.0 (d, *J* = 3 Hz) ppm; *p*-XRD (Cu-Kα): 7.0, 7.3, 7.4, 9.7, 11.2, 11.8, 12.2, 12.8, 14.2, 14.7, 14.8, 15.3, 15.6, 16.1, 17.5, 18.1, 18. 8, 19.56, 20.4, 21.2, 21.8, 22.3, 22.8, 23.2, 24.4, 24.5, 24.8, 25.4, 25.8, 26.5, 26. 8, 26.9, 27.2, 27.5, 27. 9, 28.8, 29.3, 29.9 ° 2θ.

#### 2.3.2. Synthesis of Tenofovir Alafenamide Hydrochloride (TA HCl)

A mixture of tenofovir alafenamide (360 mg, 0.76 mmol) in acetonitrile (9 mL) was heated until complete dissolution and then cooled to room temperature. The obtained solution was put in a closed chamber saturated with hydrochloric gas (prepared by addition of concentrated hydrochloric acid into concentrated sulfuric acid) and stirred. Formed fine precipitate was filtered off by centrifugation on an Amicon Ultra 100K filter at 5000 rpm, washed with acetonitrile and dried under reduced pressure at 40 °C to obtain tenofovir alafenamide hydrochloride as fine white crystalline powder. Yield: 358 mg (92% of theory). DSC (10 °K/min, two endotherms observed): 141.93 °C onset, 146.20 °C peak, 158.81 °C onset, 163.78 °C peak (literature data [117]: 144.208 onset, 146.928 °C peak, 160.025 °C onset, 163.204 °C peak); ATR-FTIR: 464, 504, 530, 583, 614, 653, 690, 715, 747, 763, 813, 898, 918, 1005, 1025, 1097, 1154, 1209, 1313, 1351, 1374, 1412, 1464, 1491, 1515, 1594, 1698, 1732, 2980, 3066 cm^-1^; ^1^H NMR (DMSO-*d_6_*, 400 MHz): *δ* 1.08−1.14 (m, 12H), 3.75−3.91 (m, 3H), 3.99 (m, 1H), 4.23 (dd, 1H, *J* = 14.4, 6.6 Hz), 4.40 (dd, 1H, *J* = 14.4, 3.5 Hz), 4.81 (sept, 1H, *J* = 6.2 Hz), 5.63 (dd, 1H, *J* = 12.1, 10.5 Hz), 7.04 (m, 2H), 7.13 (m, 1H), 7.30 (m, 2H), 8.43 (s, 1H), 8.46 (s, 1H) ppm; ^13^C-NMR (DMSO-*d_6_*, 100 MHz, proton decoupled): *δ* 16.8, 20.5 (d, *J* = 5 Hz), 21.6, 47.7, 49.2, 64.4 (d, *J* = 155 Hz), 68.1, 75.4 (d, *J* = 12 Hz), 118,0, 120.7, 124.6, 129.7, 144.6, 145.6, 149.0, 150.4, 150.8, 173.0 (d, *J* = 4 Hz) ppm; *p*-XRD (Cu-Kα): 7.0, 8.5, 9.1, 9.9, 10. 5, 10.9, 12.1, 13.4, 14.0, 14.8, 16.3, 16.9, 17.1, 17.5, 18.2, 189.0, 19.5, 20.3, 20.8, 21.0, 22.0, 22.4, 23.1, 23.5, 24.2, 24.4, 24.6, 25.4, 26.0, 26.6, 27.1, 27.7, 28.2, 28.5, 29.1, 29.4 ° 2θ (diffractogram is consistent with diffractogram from reference [117]).

#### 2.3.3. Characterization of Tenofovir Alafenamide Hemifumarate (TA HF)

Sourced TA HF, which can be prepared according to the literature procedure in [101], had the following characteristics: DSC (10 °K/min): 131.49 °C onset, 133.85 °C peak; ATR-FTIR: 481, 508, 530, 573, 584, 615, 644, 690, 723, 763, 798, 920, 977, 994, 1062, 1101, 1152, 1199, 1264, 1302, 1376, 1421, 1489, 1606, 1661, 1744, 2982, 3175 cm^−1^; ^1^H-NMR (DMSO-*d_6_*, 400 MHz): *δ* 1.05 (d, 3H, *J* = 6.2 Hz), 1.10−1.15 (m, 9 H), 3.75 (dd, 1H, *J* = 13.6, 10.1 Hz), 3.80−3.95 (m, 3H), 4.12 (dd, 1H, *J* = 14.8, 6.6 Hz), 4.26 (dd, 1H, *J* = 14.4, 3.5 Hz), 4.83 (sept, 1H, *J* = 6.2 Hz), 5.64 (dd, 1H, *J* = 11.7, 10.5 Hz), 6.62 (s, 1H), 7.03 (m, 2H), 7.12 (m, 1H), 7.21 (s, 2H), 7.27 (m, 2H), 8.09 (s, 1H), 8.13 (s, 1H) ppm; ^13^C-NMR (DMSO-*d_6_*, 100 MHz, proton decoupled): *δ* 16.8, 20.5 (d, *J* = 5 Hz), 21.6, 21.6, 47.0, 49.3, 64.3 (d, *J* = 154 Hz), 68.1, 75.7 (d, *J* = 13 Hz), 118.6, 120.7 (d, *J* = 5 Hz), 124.5, 129.7, 134.2, 141.6, 150.0, 150.4 (d, *J* = 8 Hz), 152.6, 156.1, 166.2, 173.1 (d, *J* = 4 Hz) ppm; *p*-XRD (Cu-Kα): 6.9, 8.5, 9.7, 10.0, 11.0, 11.1, 12.0, 12.2, 13.8, 14.0, 14.7, 14.8, 15.5, 15.8, 16.2, 16.6, 17.1, 17.6, 18.0, 18.3, 18.6, 19.6, 20.2, 20.8, 21.1, 21.4, 21.6, 22.0, 22.5, 23.0, 23.1, 23.3, 24.1, 24.5, 24.8, 25.0, 25.3, 25.5, 25.8, 26.5, 27.0, 27.6, 27.8, 28.3, 28.7, 29.1, 29.6 ° 2θ.

#### 2.3.4. Characterization of Tenofovir Alafenamide Monofumarate Form I (TA MF1)

Sourced TA MF1, which can be prepared according to the literature procedure in [100], had the following characteristics: DSC (10 °K/min): 120.72 °C onset, 123.11 °C peak; ATR-FTIR: 477, 500, 558, 579, 597, 633, 690, 721, 759, 783, 818, 918, 979, 1066, 1103, 1142, 1204, 1262, 1303, 1375, 1418, 1456, 1490, 1614, 1668, 1728, 2981, 3177 cm^-1^; ^1^H NMR (DMSO-*d_6_*, 400 MHz): *δ* 1.05 (d, 3H, *J* = 6.2 Hz), 1.10−1.15 (m, 9 H), 3.75 (dd, 1H, *J* = 13.2, 9.7 Hz), 3.80−3.95 (m, 3H), 4.13 (dd, 1H, *J* = 14.4, 6.6 Hz), 4.26 (dd, 1H, *J* = 14.4, 3.9 Hz), 4.83 (sept, 1H, *J* = 6.2 Hz), 5.63 (dd, 1H, *J* = 12.1, 10.5 Hz), 6.62 (s, 2H), 7.03 (m, 2H), 7.12 (m, 1H), 7.22 (s, 2H), 7.28 (m, 2H), 8.09 (s, 1H), 8.13 (s, 1H) ppm; ^13^C-NMR (DMSO-*d_6_*, 100 MHz, proton decoupled): *δ* 16.8, 20.5 (d, *J* = 5 Hz), 21.6, 21.6, 47.0, 49.2, 64.3 (d, *J* = 154 Hz), 68.1, 75.7 (d, *J* = 13 Hz), 118.6, 120.7 (d, *J* = 5 Hz), 124.5, 129.7, 134.2, 141.6, 150.0, 150.4 (d, *J* = 8 Hz), 152.6, 156.1, 166.2, 173.7 (d, *J* = 4 Hz) ppm; *p*-XRD (Cu-Kα): 5.3, 9.8, 10.4, 11.0, 11.3, 11.6, 12.3, 13.4, 13.8, 14.4, 14.9, 15.6, 15.9, 16.2, 16.6, 17.3, 17.7, 18.7, 19.0, 19.5, 20.6, 20.8, 21.2, 21.9, 22.3, 22.6, 22.9, 23.6, 23.9, 24.8, 26.6, 27.1, 27.8, 28.1, 28.9, 29.5 ° 2θ.

#### 2.3.5. Synthesis of Tenofovir Alafenamide Monofumarate Form II (TA MF2)

TA MF1 (2.00 g), prepared according to the procedure of Becker et al. [100], was suspended in acetonitrile (20 mL) and vigorously stirred at room temperature using a magnetic stirrer. The conversion of TA MF1 to TA MF2 is considered a solvent-mediated phase transformation, which takes several days to complete [118−120]. Therefore, samples for the conversion determination were taken after 6 and 10 days respectively before the crystals were finally collected by filtration after 11 days and dried under vacuum at room temperature for 17 h. Yield: 1.71 g (86% of theory).

The two intermediate samples A and B and the final product C were investigated by powder X-ray diffraction, and the results are summarized in Table 2:

The finally isolated crystals of TA MF2 (sample C, isolated after 11 days) were investigated in more detail by means of DSC, ATR-FTIR, NMR and ssNMR. The prepared TA MF2 had the following characteristics: DSC (10 °K/min): 122.43 °C onset, 124.28 °C peak; ATR-FTIR: 477, 499, 559, 579, 597, 634, 690, 722, 759, 783, 917, 981, 1067, 1103, 1149, 1204, 1262, 1304, 1375, 1417, 1491, 1614, 1668, 1729, 2981, 3177 cm^−1^; ^1^H-NMR (DMSO-*d_6_*, 400 MHz): *δ* 1.05 (d, 3H, *J* = 6.2 Hz), 1.10−1.15 (m, 9 H), 3.75 (dd, 1H, *J* = 13.6, 9.7 Hz), 3.80−3.95 (m, 3H), 4.13 (dd, 1H, *J* = 14.4, 6.2 Hz), 4.26 (dd, 1H, *J* = 14.4, 3.9 Hz), 4.83 (sept, 1H, *J* = 6.2 Hz), 5.64 (dd, 1H, *J* = 11.7, 10.5 Hz), 6.62 (s, 2H), 7.03 (m, 2H), 7.12 (m, 1H), 7.23 (s, 2H), 7.28 (m, 2H), 8.09 (s, 1H), 8.12 (s, 1H) ppm; ^13^C NMR (DMSO-*d_6_*, 100 MHz, proton decoupled): *δ* 16.8, 20.5 (d, *J* = 5 Hz), 21.6, 21.6, 47.0, 49.3, 64.3 (d, *J* = 155 Hz), 68.1, 75.7 (d, *J* = 13 Hz), 118.6, 120.7 (d, *J* = 5 Hz), 124.5, 129.7, 134.2, 141.6, 150.0, 150.4 (d, *J* = 8 Hz), 152.6, 156.1, 166.2, 173.8 (d, *J* = 4 Hz) ppm; *p*-XRD (Cu-Kα): 5.6, 7.3, 9.4, 10.1, 10.9, 11.4, 12.2, 13.0, 14.0, 14.4, 14.7, 15.1, 15.5, 16.9, 17.5, 17.8, 18.6, 18.8, 19.2, 19.6, 20.5, 21.0, 21.3, 21.5, 21.8, 22.7, 23.9, 25.4, 26.0, 26.6, 28.4 ° 2θ.

#### 2.3.6. Synthesis of Tenofovir Alafenamide Monofumarate Form III (TA MF3)

A mixture of TA MF1 (4.0 g, 8.3 mmol), prepared according to the procedure of Becker et al. [100], fumaric acid (1.0 g, 8.0 mmol) and isobutanol (40 mL) was heated to reflux temperature, whereupon a solution was obtained. The solution was filtered while hot and allowed to cool to room temperature. The obtained mixture was further stirred at room temperature for about 16 h before the crystals were collected by filtration and air-dried for 2 h to obtain the crystalline tenofovir alafenamide form S. Yield: 4.2 g (81% of theory). Tenofovir alafenamide monofumarate form S (0.5 g, prepared according to the above procedure) was stored under vacuum (20−30 mbar) at a temperature of 110 °C for 6 h, whereupon TA MF3 was obtained quantitatively. The recovered crystals of TA MF3 were investigated in more detail by means of DSC, ATR-FTIR, NMR and ssNMR. The prepared TA MF3 had the following characteristics: DSC (10 °K/min): 120.55 °C onset, 123.13 peak; ATR-FTIR: 477, 499, 560, 579, 596, 617, 634, 690, 722, 758, 782, 818, 917, 988, 1067, 1104, 1150, 1182, 1203, 1262, 1303, 1374, 1417, 1491, 1614, 1668, 1692, 1729, 2459, 2980, 3176 cm^−1^; ^1^H-NMR (DMSO-*d_6_*, 400 MHz): δ 1.05 (d, 3H, *J* = 5.8 Hz), 1.10−1.16 (m, 9 H), 3.75 (dd, 1H, *J* = 13.2, 9.7 Hz), 3.80−3.95 (m, 3H), 4.13 (dd, 1H, *J* = 14.4, 6.6 Hz), 4.25 (dd, 1H, *J* = 14.4, 3.5 Hz), 4.83 (sept, 1H, *J* = 6.2 Hz), 5.64 (t, 1H, *J* = 11.7 Hz), 6.62 (s, 2H), 7.03 (m, 2H), 7.12 (m, 1H), 7.23 (s, 2H), 7.28 (m, 2H), 8.10 (s, 1H), 8.13 (s, 1H) ppm; ^13^C NMR (DMSO-*d_6_*, 100 MHz, proton decoupled): δ 16.83, 20.57 (d, *J* = 5 Hz), 21.61, 21.64, 47.04, 49.26, 64.64.32 (d, *J* = 155 Hz), 68.10, 75.69 (d, *J* = 13 Hz), 118.58, 120.74 (d, *J* = 5 Hz), 124.55, 129.68, 134.20, 141.61, 149.98, 150.53 (d, *J* = 8 Hz), 152.60, 156.15, 166.19, 173.86 (d, *J* = 4 Hz) ppm; *p*-XRD (Cu-Kα): 5.4, 5.6, 7.3, 9.4, 9.8, 10.2, 10.6, 11.2, 11.6, 12.2, 12.6, 13.3, 14.2, 14.6, 15.1, 16.4, 17.0, 17.2, 17.7, 18.1, 18.8, 19.2, 19.6, 20.2, 20.6, 20.9, 21.4, 21.6, 21.9, 22.6, 23.7, 24.5, 24.9, 25.3, 25.9, 26.2, 26.7, 27.7, 28.3, 28.7, 29.6 ° 2θ.

## 3. Results

### 3.1. Synthesis of Tenofovir Alafenamide Derivatives

**Figure 2:**
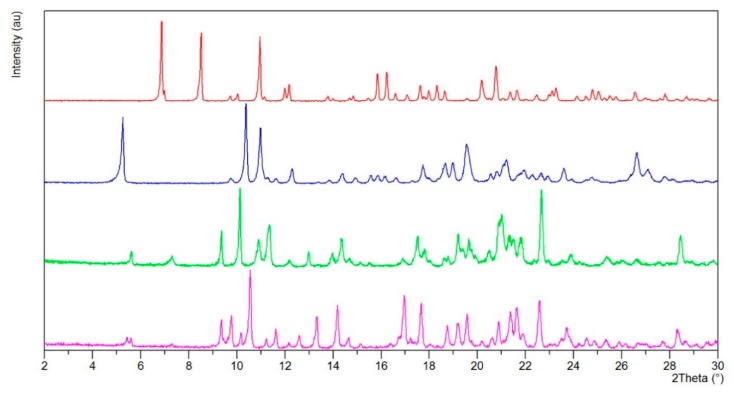
Powder X-ray diffraction patterns of tenofovir alafenamide fumarate derivatives. Red: TA HF; Blue: TA MF1; Green: TA MF2; Magenta: TA MF3.

In order to get better insights into the solid state properties of tenofovir alafenamide derivatives, we prepared TA and TA HCl in addition to the well-known TA MF1 [100] and TA HF [101] derivatives. TA was prepared in a 90% yield from TA HF by reaction with excess sodium hydrogen carbonate in water, extraction into dichloromethane and subsequent precipitation by *tert*-butyl methyl ether. TA HCl was prepared in a 92% yield by reaction of TA in acetonitrile with gaseous HCl and subsequent isolation by filtration. In addition, we were able to prepare two new tenofovir alafenamide monofumarate forms II (TA MF2) and III (TA MF3) [102]. A novel form TA MF2 was obtained via solvent-mediated phase transformation [118,119,120] by slurrying the TA MF1 in acetonitrile at ambient temperature for 11 days. Moreover, when TA MF1 in isobutanol was heated to reflux in the presence of fumaric acid, followed by cooling to ambient temperature where stirring was continued for 16 hours, a new tenofovir alafenamide form S (TA S) was obtained in a 81% yield. Exposure of TA S to vacuum (20–30 mbar) at 110 °C for 6 hours provided a novel form TA MF3, which was isolated in a quantitative yield. Both novel forms TA MF2 and TA MF3 [102] have distinctly different *p*-XRD peak patterns when compared to the previously known TA HF [101] and FA MF1 [100] (Figure 2).

### 3.2. X-Ray Single Crystal Determination

**Figure 3:**
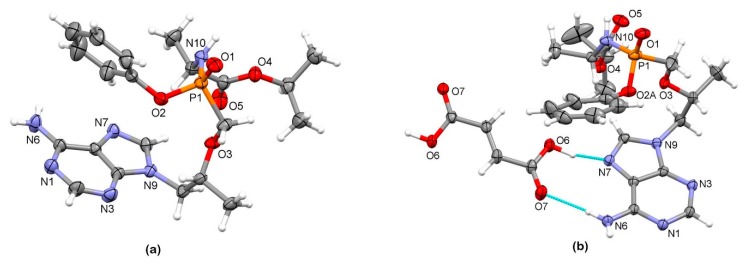
Thermal ellipsoid figures of (**a**) TA and (**b**) TA HF drawn at the 30% probability level. The asymmetric unit of TA HF contains one tenofovir alafenamide molecule and half of a molecule of fumaric acid. The disorder of the phenyl and isopropyl groups in TA HF is omitted for clarity. Hydrogen bonds are drawn with dashed blue lines.

We were able to obtain crystals suitable for the X-ray structural analysis of TA and TA HF (Figure 3a–b). The crystallographic data are listed in Table 1. Compound TA crystallizes in the orthorhombic space group *P*2_1_2_1_2_1_ with one molecule of tenofovir alafenamide in an asymmetric unit.

**Figure 4:**
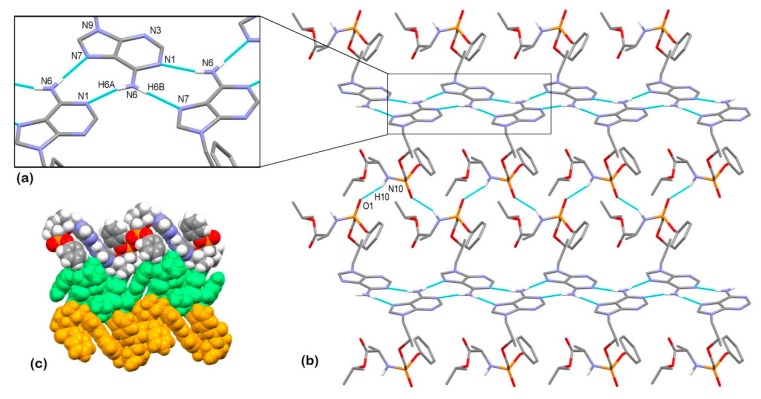
Hydrogen bond architecture in the TA crystal. (**a**) The interactions involving adenine moieties; (**b**) Wavy layered structure along the *b* axis; (**c**) Packing of layers along the *c* axis (arbitrary colors). Hydrogen bonds are drawn with dashed blue lines. Hydrogen atoms not involved in the shown motif have been omitted for clarity.

The supramolecular structure of TA is achieved through a series of hydrogen bondings. The adenine moieties of adjacent molecules are connected in a chain through a combination of N6–H6a···N1^i^ and N6–H6b···N7^ii^ hydrogen bonding between the amine NH_2_ group as a hydrogen-bond donor and aromatic N atoms of the adjacent adenine moieties as hydrogen-bond acceptors, forming a R^2^_2_(9) ring motif [121] (symmetry codes: (i) ½ + *x*, ½ − *y*, 1 – *z*; (ii) −½ + *x*, ½ − *y*, 1 − *z*). The chains are further connected into a wavy layer through N10–H10···O1^iii^ hydrogen bonding between the amide group as a hydrogen-bond donor and the oxygen atom of the P=O group as a hydrogen-bond acceptor (symmetry code: (iii) ½ + *x*, 3/2 − *y*, 1 − *z*) (Figure 4a–c).

**Figure 5:**
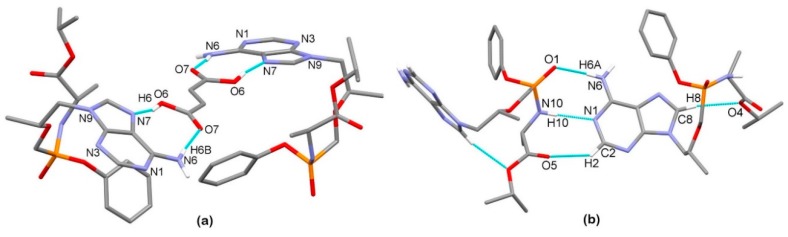
Hydrogen bond architecture in the TA HF co-crystal. (**a**) Hydrogen bonding between both carboxylic groups of fumaric acid and two adjacent tenofovir alafenamide molecules; (**b**) Hydrogen bonding between adjacent tenofovir alafenamide molecules and intramolecular hydrogen bonding. Hydrogen bonds are drawn with dashed blue lines. Hydrogen atoms not involved in the shown motif have been omitted for clarity. The disorder of the isopropyl and phenyl groups is omitted for clarity.

The compound TA HF crystallizes in the tetragonal space group *P*4_2_2_1_2. The asymmetric unit is composed of one molecule of tenofovir alafenamide and half of a molecule of fumaric acid due to the 2-fold rotation axis (Figure 3b). The compound crystallizes as a co-crystal since no proton transfer from the fumaric acid to TA is observed, and the solid phase is a 2:1 co-crystal of tenofovir alafenamide and fumaric acid (Figure 5a). Two TA molecules are linked at each end of the fumaric acid through O6–H6···N7 hydrogen bonding between the –COOH group as a hydrogen-bond donor and the aromatic N7 atom of the adenine moiety of TA in cooperation with N6–H6b···O7 bonding between the amine NH_2_ group of the adenine moiety as a hydrogen-bond donor and the carbonyl oxygen of the –COOH group as a hydrogen-bond acceptor, forming a R^2^_2_(9) ring motif. These 2:1 complexes are further connected into a 3D supramolecular structure through a series of hydrogen bondings. The TA molecule is connected to the adjacent TA molecule through a combination of N6–H6a···O1^i^ and N10^i^–H10^i^···N1 hydrogen bonding with the NH_2_ group of the adenine moiety and the amide group both acting as hydrogen-bond donors and the oxygen atom of the P=O group and aromatic N1 atom of the adenine moiety as hydrogen-bond acceptors, forming a R^2^_2_(8) ring motif. These hydrogen bondings are supported by C2–H2···O5^i^ bonding between the CH group of the adenine moiety as a hydrogen-bond donor and the O5 carbonyl atom of the isopropoxycarbonyl moiety, forming an additional R^2^_2_(8) ring motif (symmetry code: (i) 3/2 − *y*, ½ + *x*, ½ + *z*) (Figure 5b).

### 3.3. Solid-State Nuclear Magnetic Resonance Analysis

Preliminary NMR studies on tenofovir alafenamide included ^1^H echo MAS NMR spectra, which showed signals between *δ_H_* 15 and 20 ppm for tenofovir alafenamide hydrochloride salt, monofumarate and hemifumarate species (Figures S24–S25). The observed ^1^H-NMR signals suggest the presence of hydrogen bonds. On the other hand, tenofovir alafenamide showed no signals in the region above *δ_H_* 12 ppm. The focus of the NMR characterization was on ^15^N experiments.

**Figure 6:**
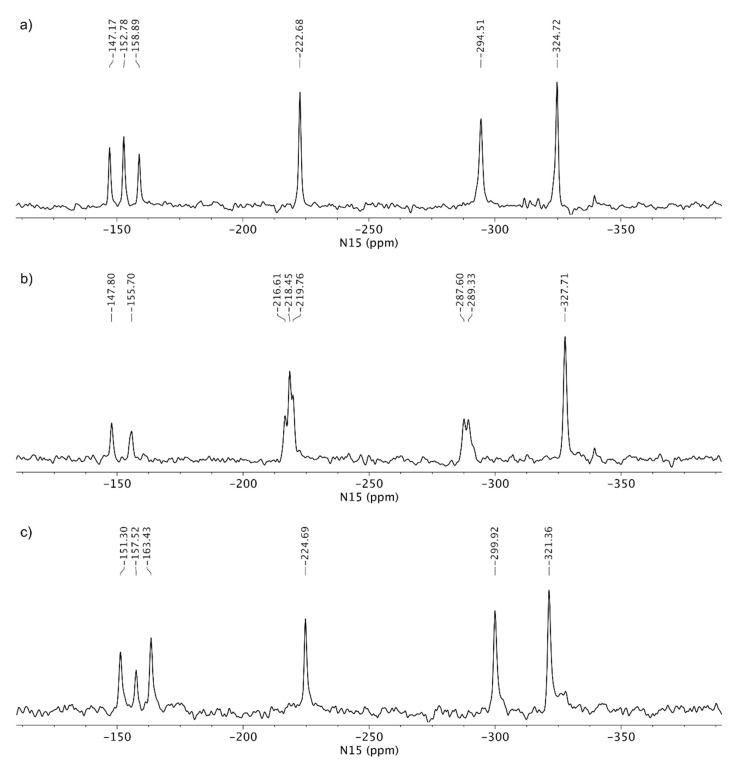
^15^N CP MAS NMR (^15^N cross-polarization magic angle spinning nuclear magnetic resonance) spectra of (**a**) TA, (**b**) TA HCl and (**c**) TA HF.

Tenofovir alafenamide free base showed four groups of signals in the ^15^N CP-MAS NMR spectrum (Figure 6a). The lowest NMR chemical shift was assigned to phosphoramidate nitrogen (*δ_N_* −324.7 ppm), followed by aminopurine nitrogen at *δ_N_* −294.5 ppm. The alkylated nitrogen N9 showed a chemical shift at *δ_N_* −222.7 ppm, while the signals between *δ_N_* −147.2 and −158.9 ppm were attributed to the rest of the purine nitrogen atoms (N1, N3 and N7, Figure 6a). Tenofovir alafenamide free base (TA) presents reference substance for non-protonated species of tenofovir alafenamide. On the contrary, tenofovir alafenamide hydrochloride salt (TA HCl) serves as reference for the protonated form. The comparison of ^15^N CP-MAS NMR spectra for TA and TA HCl showed only two ^15^N signals in the region between *δ_N_* −147 and −158 ppm (three were observed for free base). In addition, multiple ^15^N signals were detected in the region between *δ_N_* −216 and -220 ppm (only one was observed for TA). The observed differences for TA and TA HCl suggest that protonation occurred on one of the N1, N3 or N7 purine nitrogen atoms, which is consistent with previous literature reports on adenosinium picrate [104]. The protonation of the purine nitrogen atom showed a significant NMR chemical shift change of ca. 60 ppm. Furthermore, some of the NMR signals in the ^15^N CP-MAS spectrum of TA HCl are doubled, which suggests that more than one molecule is present per asymmetric unit (Figure 6b). TA HF showed ^15^N chemical shifts similar to TA; therefore, the protonation of TA by hemifumarate species was excluded (Figure 6c). Three different samples of monofumarate forms showed ^15^N-NMR signals in regions very similar to those of reference substances of TA and TA HCl (Figure S26).

**Figure 7:**
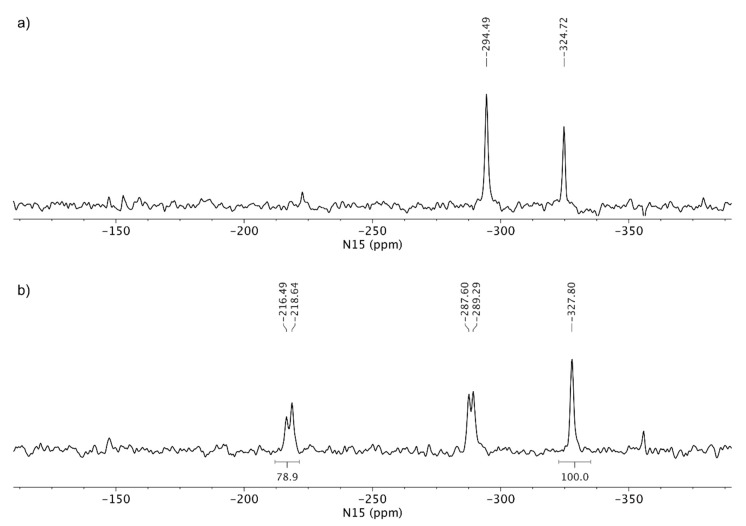
^15^N LG-CP MAS NMR (Lee-Goldburg cross-polarization magic angle spinning nuclear magnetic resonance) spectra of (**a**) TA and (**b**) TA HCl. Both spectra were acquired using a relaxation delay of 2 s and ca. 40,000 scans. The relative integral value of protonated purine nitrogen is reported with respect to the integral region of amine nitrogen, which was arbitrarily set at 100.

The next step of characterization involved ^15^N LG-CP MAS NMR spectra, as shown in Figure 7. A short excitation time of 200 μs was used to transfer the polarization to nitrogen atoms, which resulted in the selective detection of protonated nitrogen atoms. TA showed two signals in the ^15^N LG-CP MAS NMR spectra: phosphoramidate nitrogen at *δ_N_* −324.7 ppm and aminopurine nitrogen at *δ_N_* −294.5 ppm. For comparison, TA HCl showed an additional set of signals in the region between *δ_N_* −216 and −220 ppm, which confirms the protonation of one of the nitrogen atoms (Figure 7).

**Figure 8:**
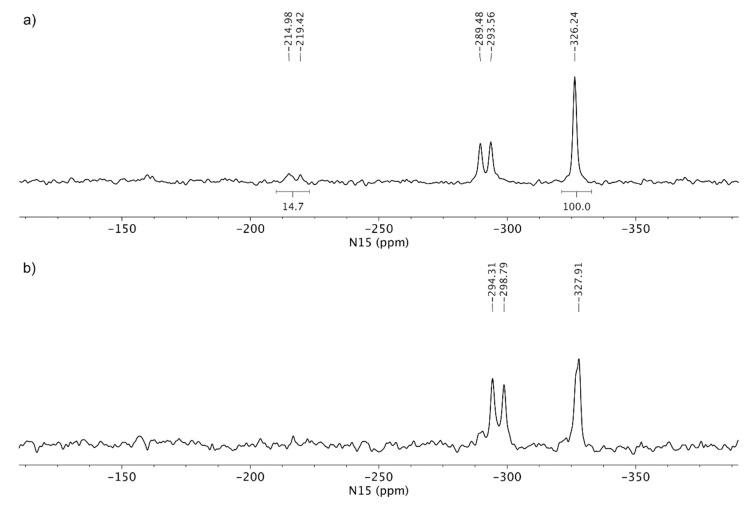
^15^N LG-CP MAS NMR spectra of (**a**) TA MF1 and (**b**) TA MF3. Both spectra were acquired using a relaxation delay of 1 s and ca. 200,000 scans. The relative integral value of protonated purine nitrogen is reported with respect to the integral region of amine nitrogen, which was arbitrarily set at 100.

^15^N LG-CP MAS NMR spectra of the tenofovir alafenamide monofumarate samples TA MF1 and TA MF3 are shown in Figure 8. TA MF3 showed signals at *δ_N_* −327.9 ppm (phosphoramidate nitrogen), as well as two signals at *δ_N_* −294.3 and −298.8 ppm (aminopurine nitrogen), which are in good agreement with the NMR chemical shifts of the TA form, which suggests that no protonation occurred in the case of TA MF3 and that the substance was obtained as a pure co-crystal structure. Interestingly, an additional set of weak signals was observed for TA MF1 in the region between *δ_N_* −214 and −220 ppm, which were attributed to protonated species of tenofovir alafenamide monofumarate. The comparison of the ^15^N spectral properties of TA HCl and TA MF1 allowed us to estimate that major species in TA MF1 correspond to the co-crystal form, whereas a small portion of ca. 20% represents protonated species (Figure 8). TA MF2 showed similar ^15^N LG-CP MAS NMR data as those obtained for TA MF3; therefore, it was confirmed that TA MF2 exists in a pure co-crystal structure (Figure S27). 

## 4. Discussion

In our report, we present the first overall study on tenofovir alafenamide fumarate derivatives with the aim to determine the solid-state structure of these derivatives. In our study, we have determined that TA MF2 and TA MF3 exist in addition to the well-known TA MF1 and TA HF derivatives. This makes the overall landscape of tenofovir alafenamide fumarate derivatives densely populated. The fundamental question in connection to the tenofovir alafenamide fumarate derivatives’ structure that remained to be answered was related to the continuum between the salt and co-crystal states. Indeed, the combination of tenofovir, which possesses an adenine heterocyclic core that contains nitrogen atoms (p*K*_a1_ = 4.2, p*K*_a2_ = 9.8) [103,104,105,106], and fumaric acid (p*K*_a1_ = 3.0, p*K*_a2_ = 4.4) [107] might result in complexes with no proton transfer, due to a small Δp*K*_a_ (p*K*_a_ of base - p*K*_a_ of acid) value of ca. 1.2. In order to solve this question, we first prepared the TA and TA HCl, which represent extreme states of the salt and co-crystal continuum. Both TA and TA HCl could serve as reference standards for the determination of the degree of ionization in TA HF and TA MF derivatives using ^15^N-ssNMR.

In addition, we were able to obtain crystals of TA and TA HF suitable for single crystal X-ray diffraction. Interestingly, these experiments clearly revealed that TA HF exists as a co-crystal structure.

**Figure 9:**
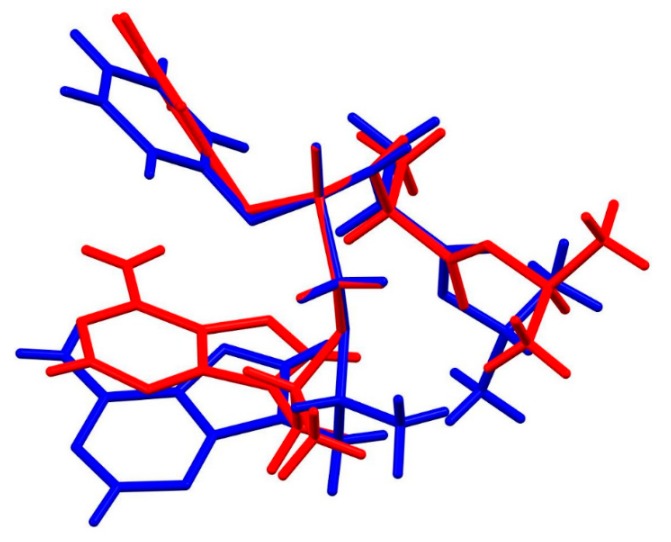
Superposition showing the difference in conformation of tenofovir alafenamide molecules in TA (red) and TA HF (blue). The disorder on the isopropyl and phenyl groups is omitted for clarity.

Moreover, the presence of fumaric acid in TA HF causes different hydrogen bonding motifs in TA HF co-crystals in comparison to TA crystals. Hydrogen bonding interactions and packing effects cause also changes in the conformation of the tenofovir alafenamide molecule. The molecular overlay shows the main difference to be in the orientation of the isopropoxycarbonyl moiety, with a N10–C13–C15–O4 torsion angle of –13.7(4)° (TA) vs. 163.7(2)° (TA HF). The difference in conformation is also present in the adenine-containing substituent, primarily due to the alteration of the P1–C12–O3–C10 torsion angle (−123.0(2)° for TA vs. –162.8(2)° for TA HF) (Figure 9); additionally, slight differences in conformation can also be observed for the phenoxy group.

**Figure 10:**
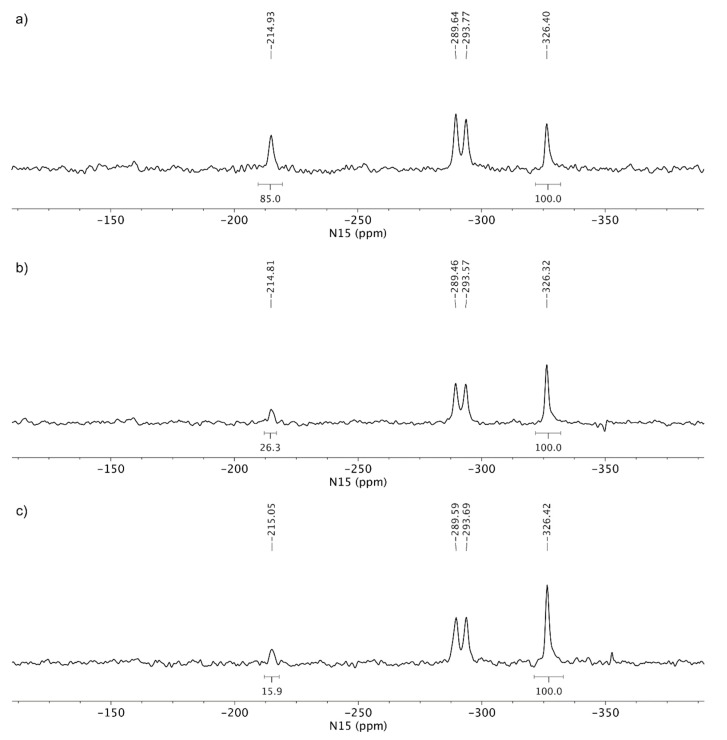
^15^N LG-CP MAS NMR spectra of different samples of TA MF1: (**a**) the majority of samples contains salt species, (**b**) the sample contains approximately 30% of salt species and (**c**) the sample contains approximately 20% of salt species. All spectra were acquired using a relaxation delay of 1 s and ca. 200,000 scans. The relative integral value of protonated purine nitrogen is reported with respect to the integral region of amine nitrogen, which was arbitrarily set at 100.

Since we could not obtain crystals of TA MF derivatives suitable for single crystal X-ray diffraction, we applied ssNMR to determine if they exist as salts, co-crystals or complexes with mixed ionized states. TA and TA HCl were chosen as reference substances for the non-protonated and protonated forms of the TA analogues, respectively. The comparison of the ^15^N CP-MAS NMR spectra of TA and TA HCl suggests that protonation occurred on one of the N1, N3 or N7 purine nitrogen atoms, where the protonation of the purine nitrogen atom showed a significant NMR chemical shift change of ca. 60 ppm. TA HF showed ^15^N chemical shifts similar to TA, hence the protonation of TA by fumaric acid species was excluded (compare Figure 6a,c). The ^15^N LG-CP MAS NMR spectra shown in Figure 7 and Figure 8 allowed for the selective detection of protonated nitrogen atoms. This type of solid-state NMR experiment clearly showed an additional protonated nitrogen atom in the region between *δ_N_* −216 and −220 ppm for TA HCl salt with respect to the TA form. TA MF2 and TA MF3 showed a high resemblance of ^15^N-NMR chemical shifts with respect to the TA form, which indicates that no protonation occurred in the case of TA MF2 or TA MF3 and that both substances were obtained as pure co-crystal structures. Interestingly, an additional set of weak signals was observed for TA MF1 in the region between *δ_N_* −214 and −220 ppm, which were attributed to protonated species of tenofovir alafenamide monofumarate. A further comparison of the ^15^N spectral properties of TA HCl and TA MF1 allowed us to estimate that the major species in the majority of the TA MF1 samples correspond to the co-crystal form, whereas a small portion of approximately 20%–30% represent protonated species (Figure 10b,c). Interestingly, we were also able to obtain a sample of TA MF1, which was composed entirely of salt species (100%) (Figure 10a). This finding indicates that the variable amount of salt species present in TA MF1 essentially depends on the process used to prepare TA MF1.

## 5. Conclusions

Our study provides the first insights into the complex solid-state chemistry of tenofovir alafenamide fumarate derivatives and provides important regulatory implications according to the FDA guideline on the regulatory classification of pharmaceutical co-crystals [108]. We have prepared two novel tenofovir alafenamide monofumarate forms: TA MF2 and TA MF3. We also report the first full characterization of the previously known TA MF1 [100] and TA HF [101], together with the new monofumarate forms TA MF2 and TA MF3 [102]. The data obtained in our study indicate that TA HF, TA MF2 and TA MF3 can exist as pure co-crystal structures, while TA MF1 was found either as a mixture of salt and co-crystal states or as a pure salt species.

## Figures and Tables

**Table 1:** Crystallographic data of tenofovir alafenamide (TA) and tenofovir alafenamide hemifumarate (TA HF).

	TA	TA HF
CCDC number	1990630	1990631
Formula	C_21_H_29_N_6_O_5_P	C_46_H_62_N_12_O_14_P_2_
*M*_r_	476.47	1069.01
*T* (K)	293(2) K	150(2) K
Crystal system	Orthorhombic	Tetragonal
Space group	*P*2_1_2_1_2_1_	*P*4_2_2_1_2
*a* (Å)	8.4062(2) Å	18.0407(2)
*b* (Å)	15.7401(3)	18.0407(2)
*c* (Å)	18.2196(4)	17.5003(2)
Volume (Å^3^)	2410.72(9)	5695.77(14)
Z	4	4
*D*_c_ (g/cm^3^)	1.313	1.247
*μ* (mm^–1^)	1.385	1.283
*F*(000)	1008	2256
Reflections collected	9201	23148
Independent reflections (*R*_int_)	4403 (0.0336)	5834 (0.0316)
Data/restraints/parameters	4403 / 3 / 312	5834 / 3 / 432
*R*, *wR*_2_ [*I* > 2σ(*I*)] *^a^*	0.0380, 0.0966	0.0383, 0.0976
*R*, *wR*_2_ (all data) *^a^*	0.0452, 0.1013	0.0430, 0.1029
GOF, *S* ^b^	1.024	1.016
Largest diff. peak/hole / e Å^−3^	0.299/−0.200	0.177/−0.369
Flack parameter	0.023(17)	−0.020(10)

*^a^ R* = ∑||*F*_o_| − |*F*_c_||/∑|*F*_o_|, *wR*_2_ = {∑[*w*(*F*_o_^2^ − *F*_c_^2^)^2^]/∑[*w*(*F*_o_^2^)^2^]}^1/2^. *^b^ S* = {∑[(*F*_o_^2^ − *F*_c_^2^)^2^]/(*n*/*p*}^1/2^, where *n* is the number of reflections and *p* is the total number of refined parameters.

**Table 2:** Monitoring of the conversion of TA MF1 to TAM F2.

Sample	Isolated	Result according to powder X-ray diffraction
A	6 days	TA MF2 + traces TA MF1
B	10 days	TA MF2
C	11 days	TA MF2

## Supplementary Materials

The following are available online at https://www.mdpi.com/1999-4923/12/4/342/s1, Figure S1: DSC thermogram of TA HF, Figure S2: DSC thermogram of TA MF1, Figure S3: DSC thermogram of TA MF2, Figure S4: DSC thermogram of TA MF3, Figure S5: overlay of DSC thermograms of TA MF1, TA MF2 and TA MF3, Figure S6: ATR-FTIR spectrum of TA, Figure S7: ATR-FTIR spectrum of TA HCl, Figure S8: ATR-FTIR spectrum of TA HF, Figure S9: ATR-FTIR spectrum of TA MF1, Figure S10: ATR-FTIR spectrum of TA MF2, Figure S11: ATR-FTIR spectrum of TA MF3, Figure S12: **^1^**H-NMR spectrum of TA, Figure S13: **^13^**C-NMR spectrum of TA, Figure S14: **^1^**H-NMR spectrum of TA HCl, Figure S15: **^13^**C-NMR spectrum of TA HCl, Figure S16: **^1^**H-NMR spectrum of TA HF, Figure S17: **^13^**C-NMR spectrum of TA HF, Figure S18: **^1^**H-NMR spectrum of TA MF1, Figure S19: **^13^**C-NMR spectrum of TA MF1, Figure S20: **^1^**H-NMR spectrum of TA MF2, Figure S21: **^13^**C-NMR spectrum of TA MF2, Figure S22: **^1^**H-NMR spectrum of TA MF3, Figure S23: **^13^**C-NMR spectrum of TA MF3, Figure S24: ^1^H echo MAS NMR spectra of TA, TA HCl and TA HF, Figure S25: ^1^H echo MAS NMR spectra of TA MF1, TA MF2 and TA MF3, Figure S26: ^15^N CP MAS NMR spectra of TA MF1, TA MF2 and TA MF3, Figure S27: ^15^N LG-CP MAS NMR spectra of TA MF1, TA MF2 and TA MF3, Table S1: detailed *p*-XRD data for TA HF, TA MF1, TA MF2 and TA MF3. CCDC 1990630 and 1990631 contain the supplementary crystallographic data for this paper. These data can be obtained free of charge via www.ccdc.cam.ac.uk/data_request/cif or by emailing data_request@ccdc.cam.ac.uk or by contacting The Cambridge Crystallography Data Centre, 12 Union Road, Cambridge CB2 1EZ, UK; fax: +44 1223 336033.
