# Supplementary material for: Co-crystals, Salts or Mixtures of Both? The Case of Tenofovir Alafenamide Fumarates
Source: Pharmaceutics. 2020 Apr 10;12(4):342. doi: 10.3390/pharmaceutics12040342 (PMC7238255; doi:10.3390/pharmaceutics12040342)
Supplement: Supplementary file 1 [file pharmaceutics-12-00342-s001.pdf]

pharmaceutics

MDPI

Supplementary Materials: Co-crystals, Salts or Mixtures of Both? The Case of Tenofovir Alafenamide Fumarates

Hannes Lengauer, Damjan Makuc, Damjan Šterk, Franc Perdih, Arthur Pichler, Tina Trdan Lušin, Janez Plavec and Zdenko Časar

1. DSC thermograms

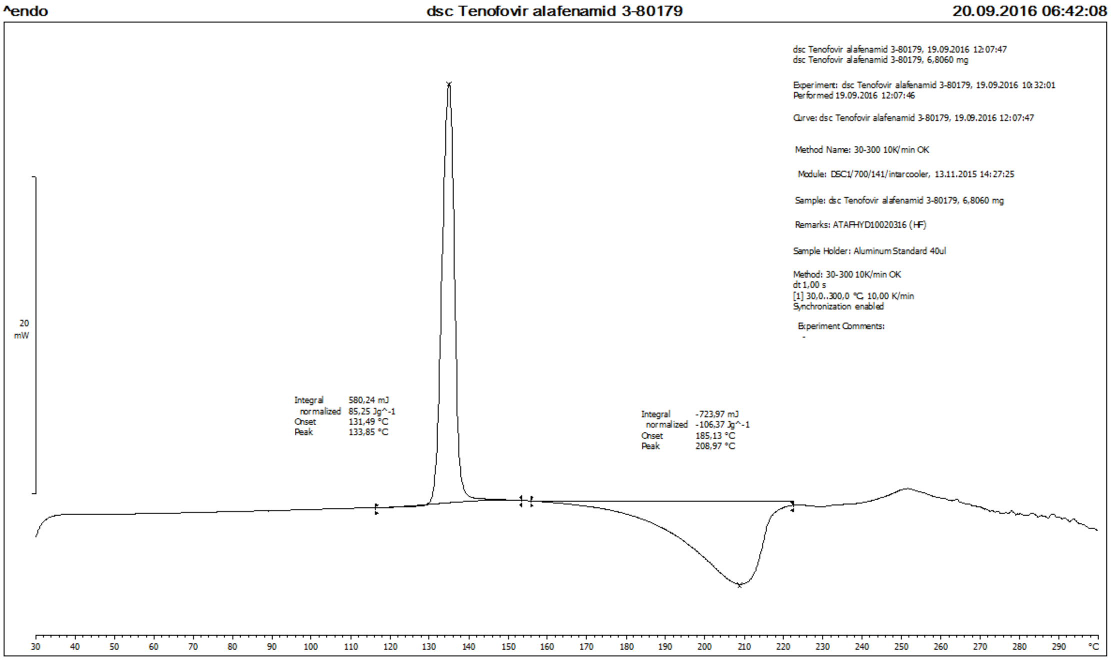

Figure S1. DSC thermogram of tenofovir alafenamide hemifumarate (TA HF).

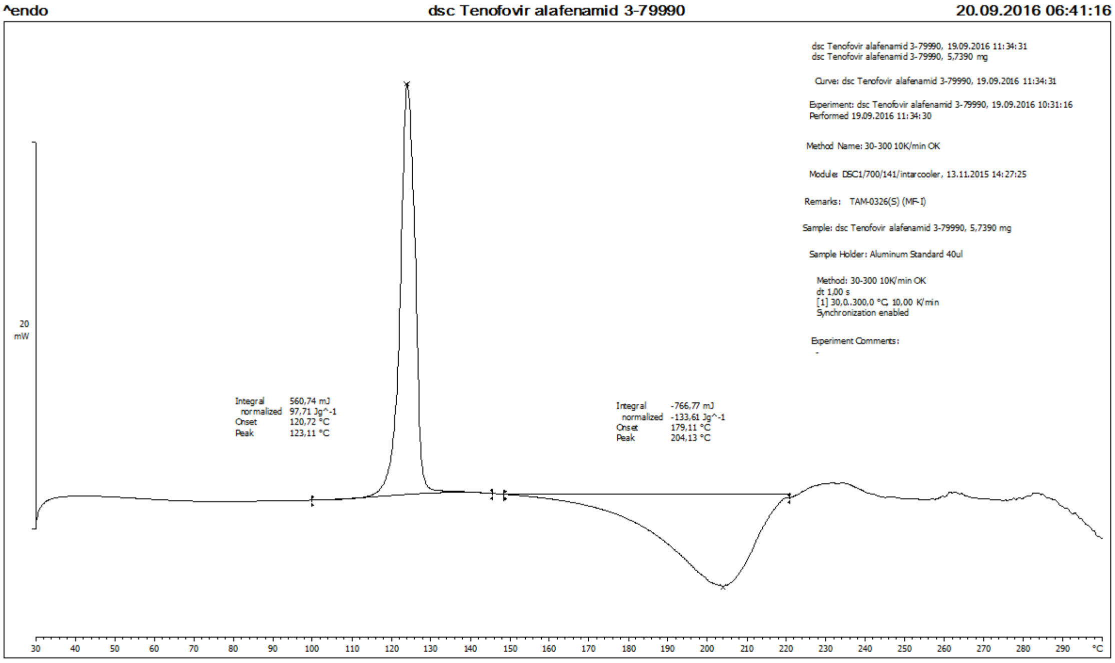

Figure S2. DSC thermogram of tenofovir alafenamide monofumarate I (TA MF1).

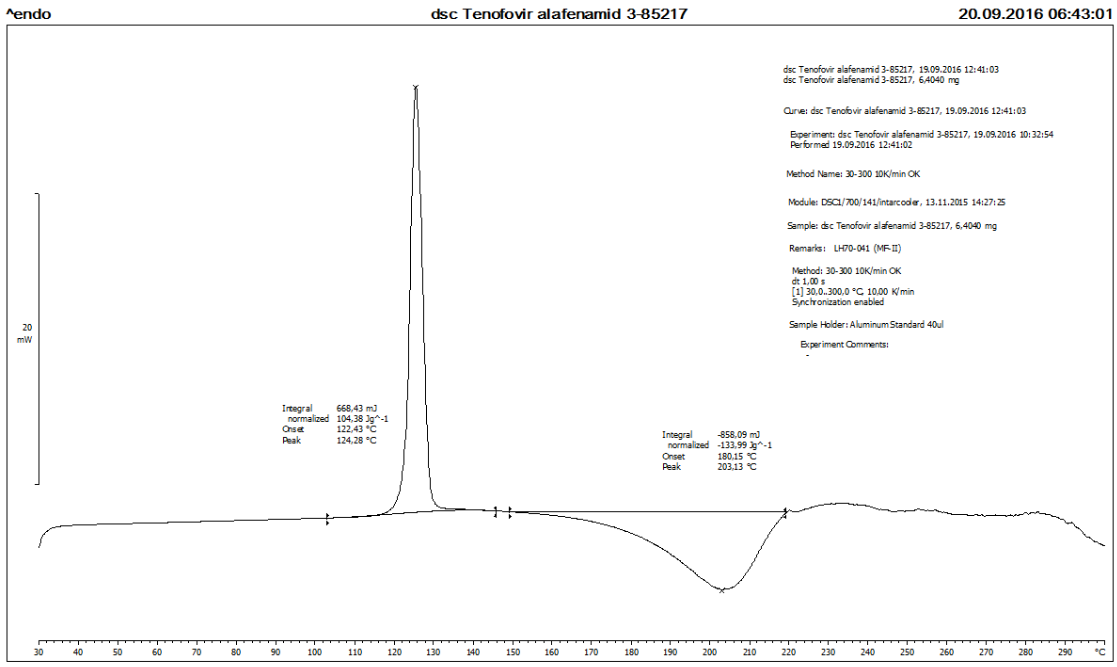

Figure S3.DSC thermogram of tenofovir alafenamide monofumarate II (TA MF2).

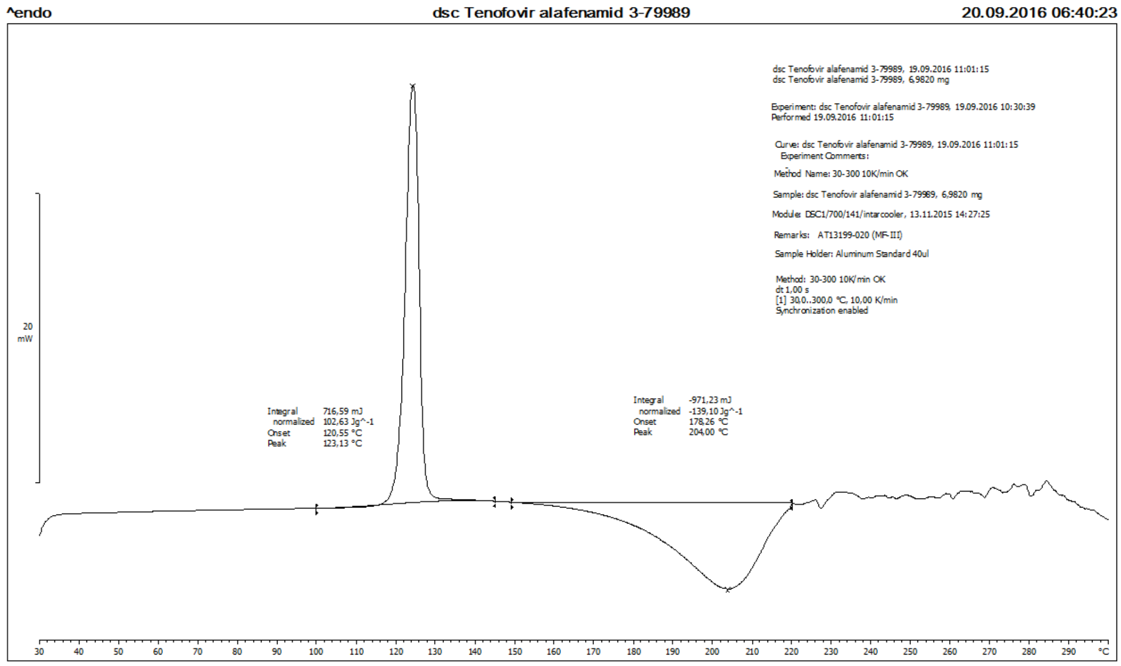

Figure S4.DSC thermogram of tenofovir alafenamide monofumarate III (TA MF3).

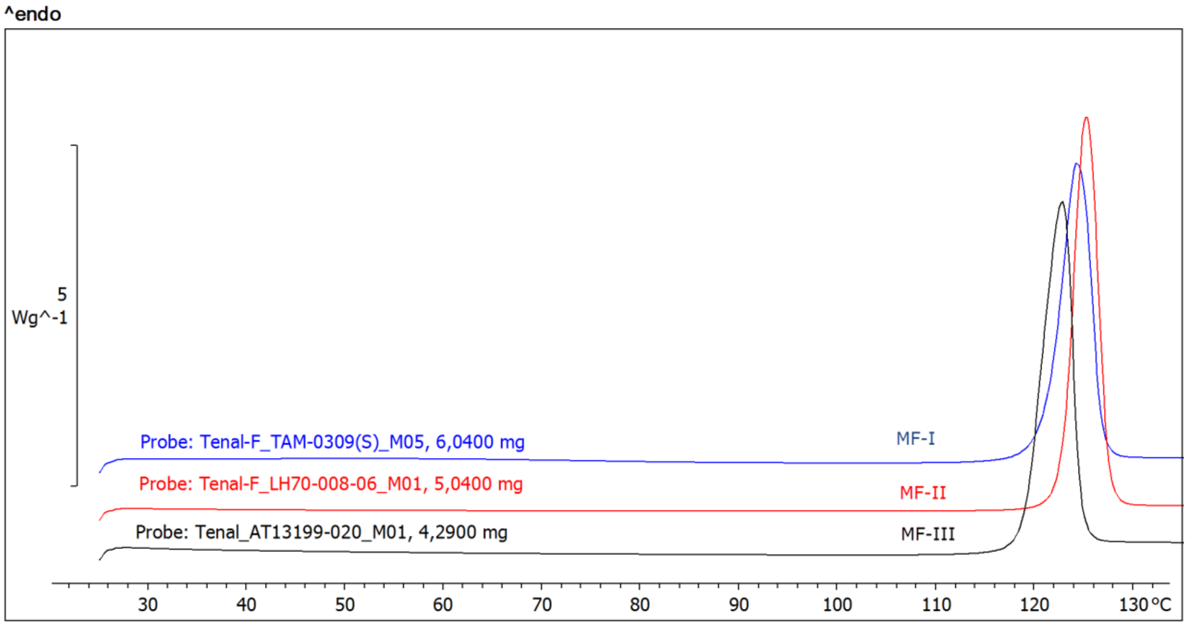

Figure S5. Overlay of DSC thermograms of tenofovir alafenamide monofumarate I (TA MF1), blue; tenofovir alafenamide monofumarate II (TA MF2), red; tenofovir alafenamide monofumarate III (TA MF3), black.

2. ATR-FTIR spectra

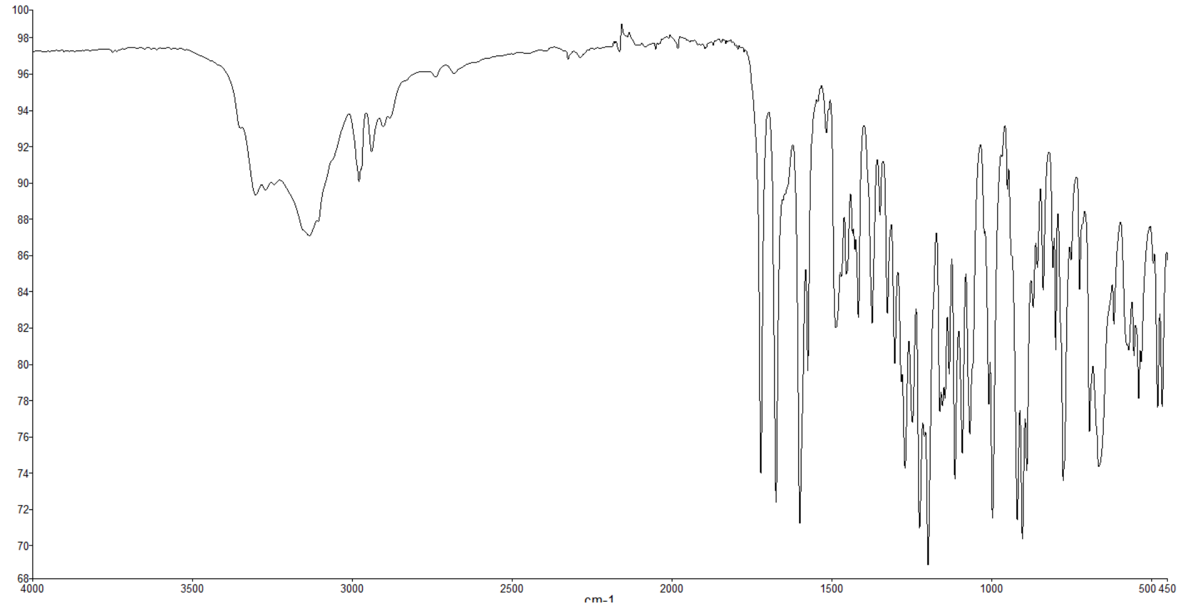

Figure S6.ATR-FTIR spectrum of tenofovir alafenamide (TA).

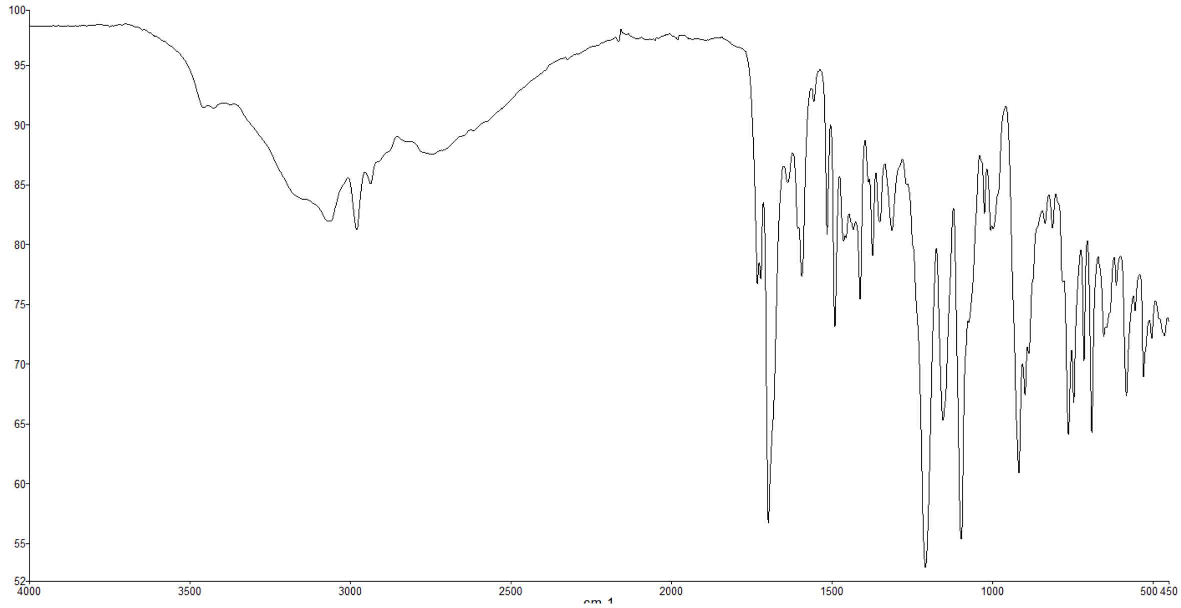

Figure S7.ATR-FTIR spectrum of tenofovir alafenamide hydrochloride (TA HCl).

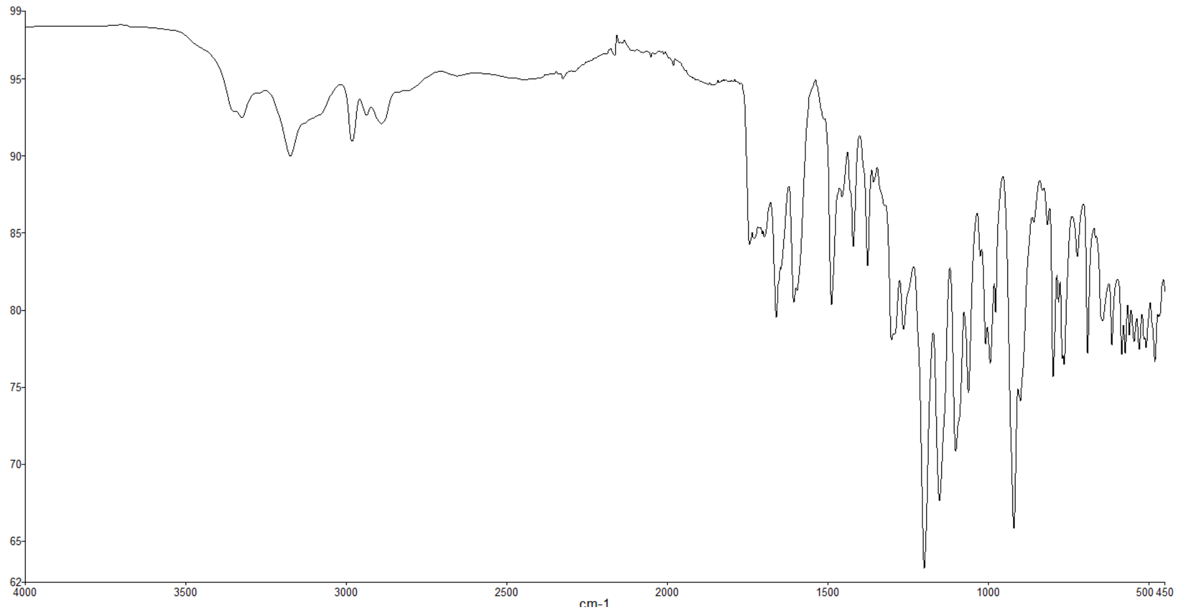

Figure S8.ATR-FTIR spectrum of tenofovir alafenamide hemifumarate (TA HF).

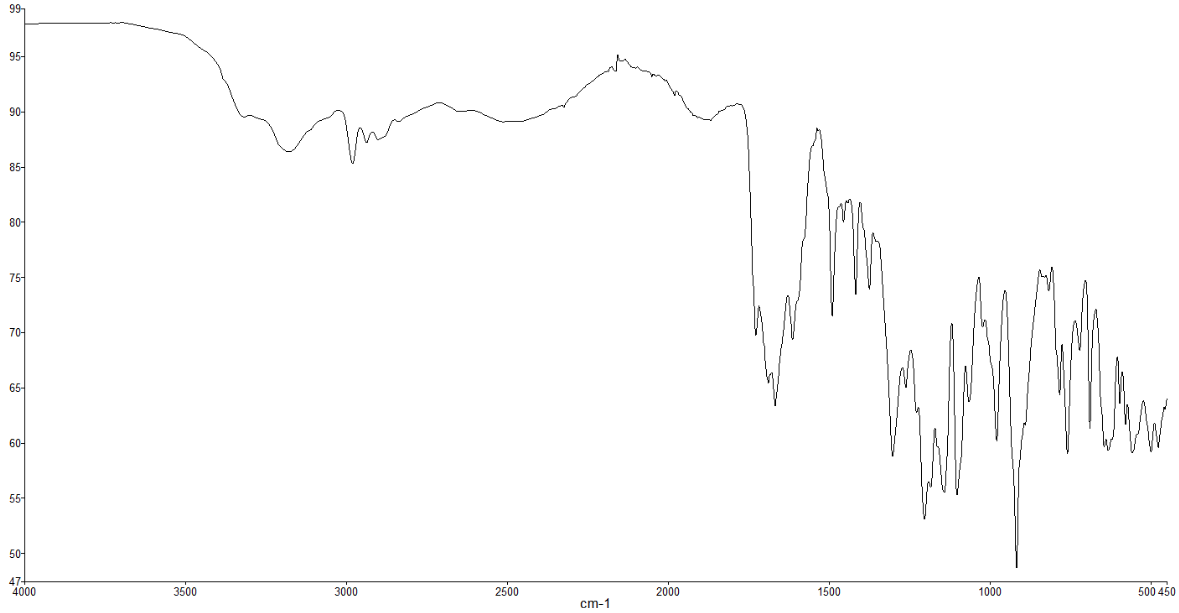

Figure S9.ATR-FTIR spectrum of tenofovir alafenamide monofumarate I (TA MF1).

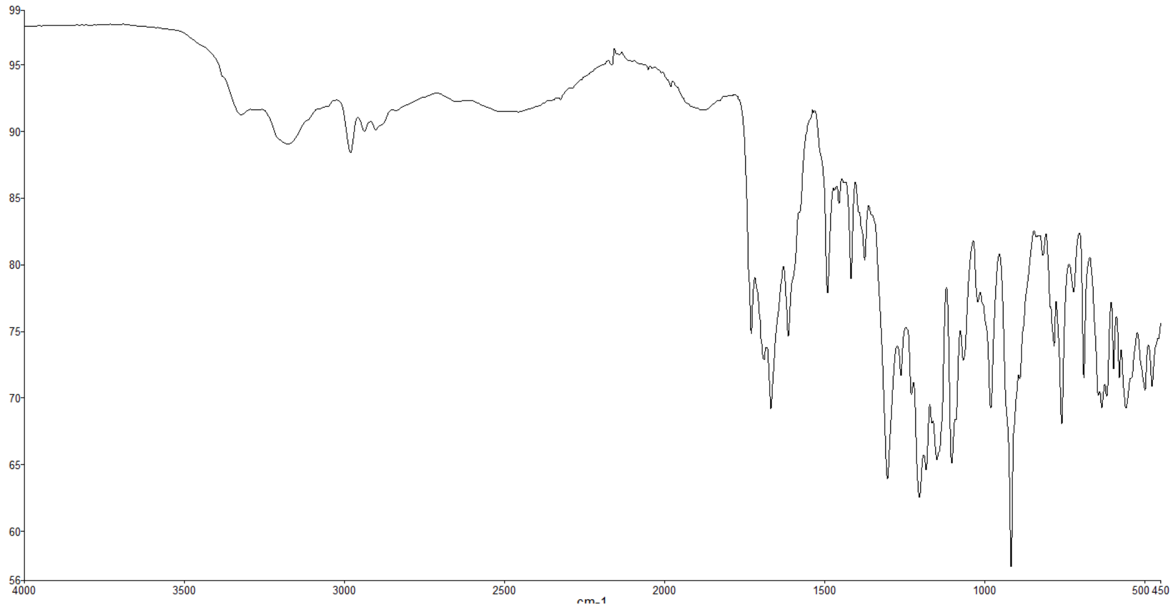

Figure S10.ATR-FTIR spectrum of tenofovir alafenamide monofumarate II (TA MF2).

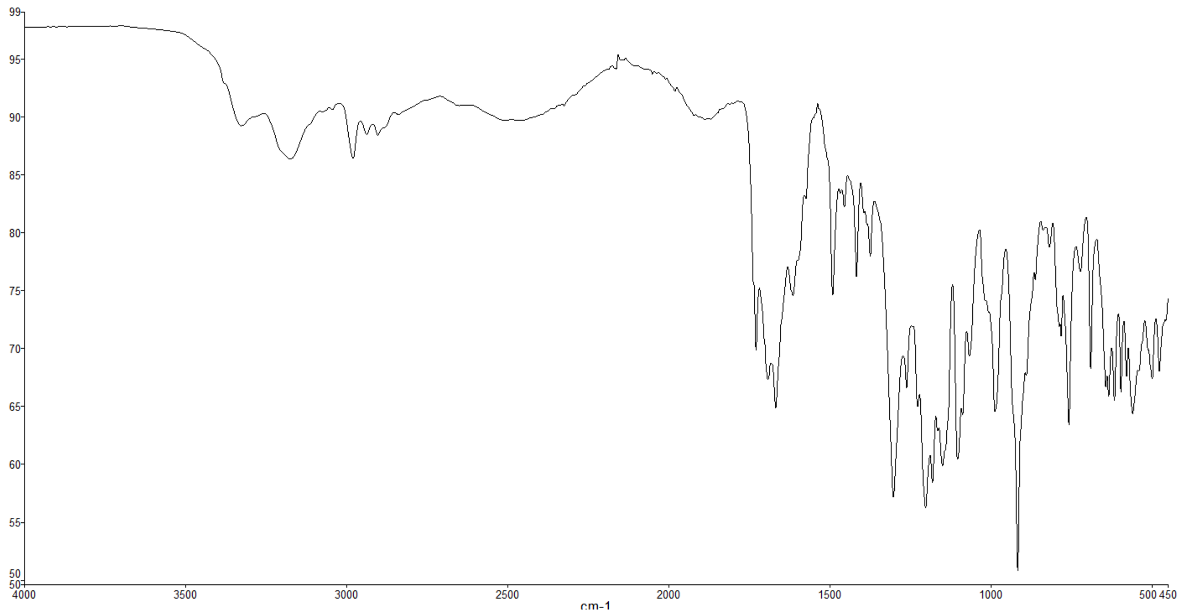

Figure S11.ATR-FTIR spectrum of tenofovir alafenamide monofumarate III (TA MF3).

3. Solution ^{1}H- and ^{13}C-NMR spectra of tenofovir alafenamide derivatives

3.1. Tenofovir alafenamide (TA)

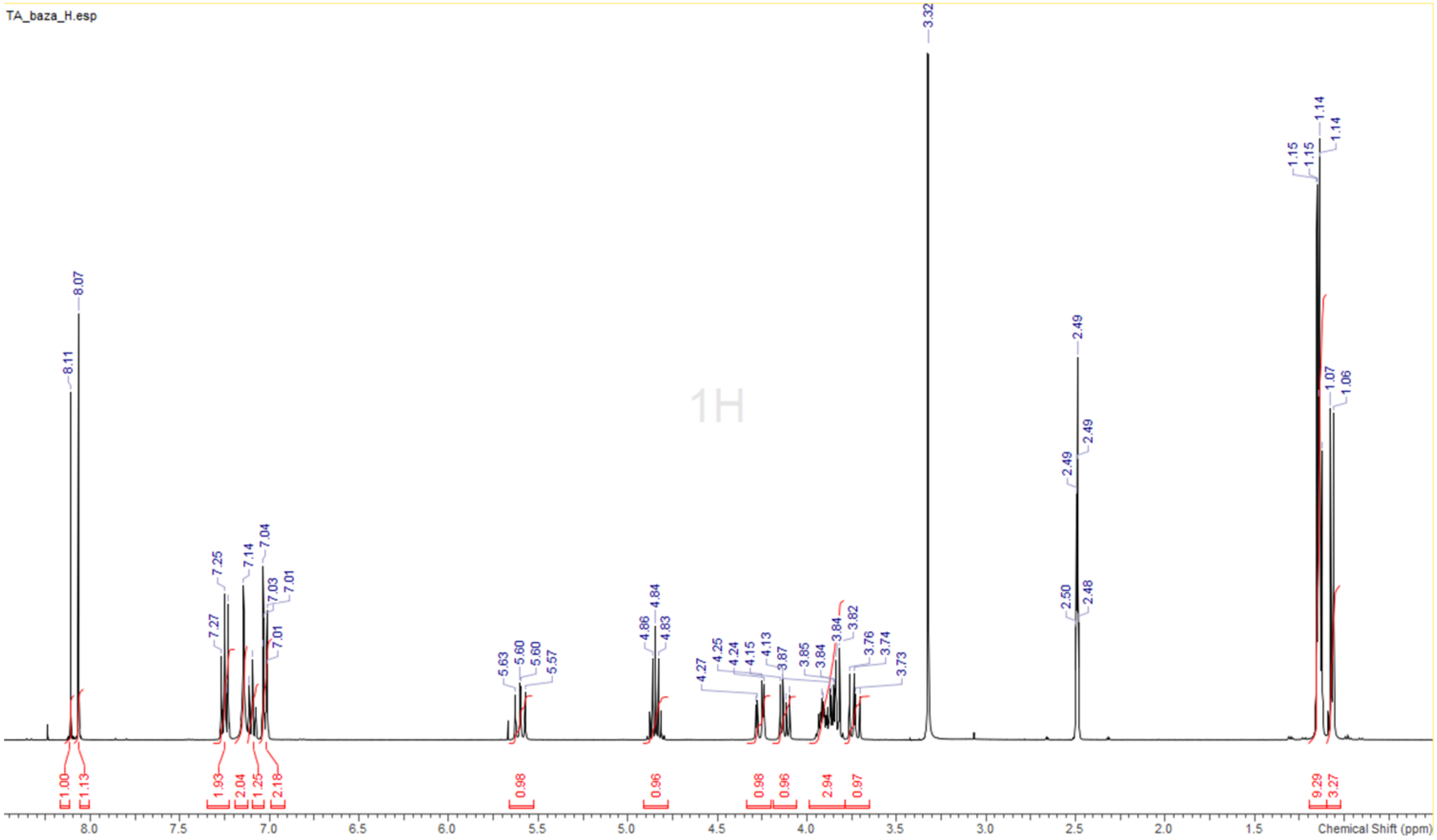

Figure S12. ^{1}H-NMR spectrum of tenofovir alafenamide (TA).

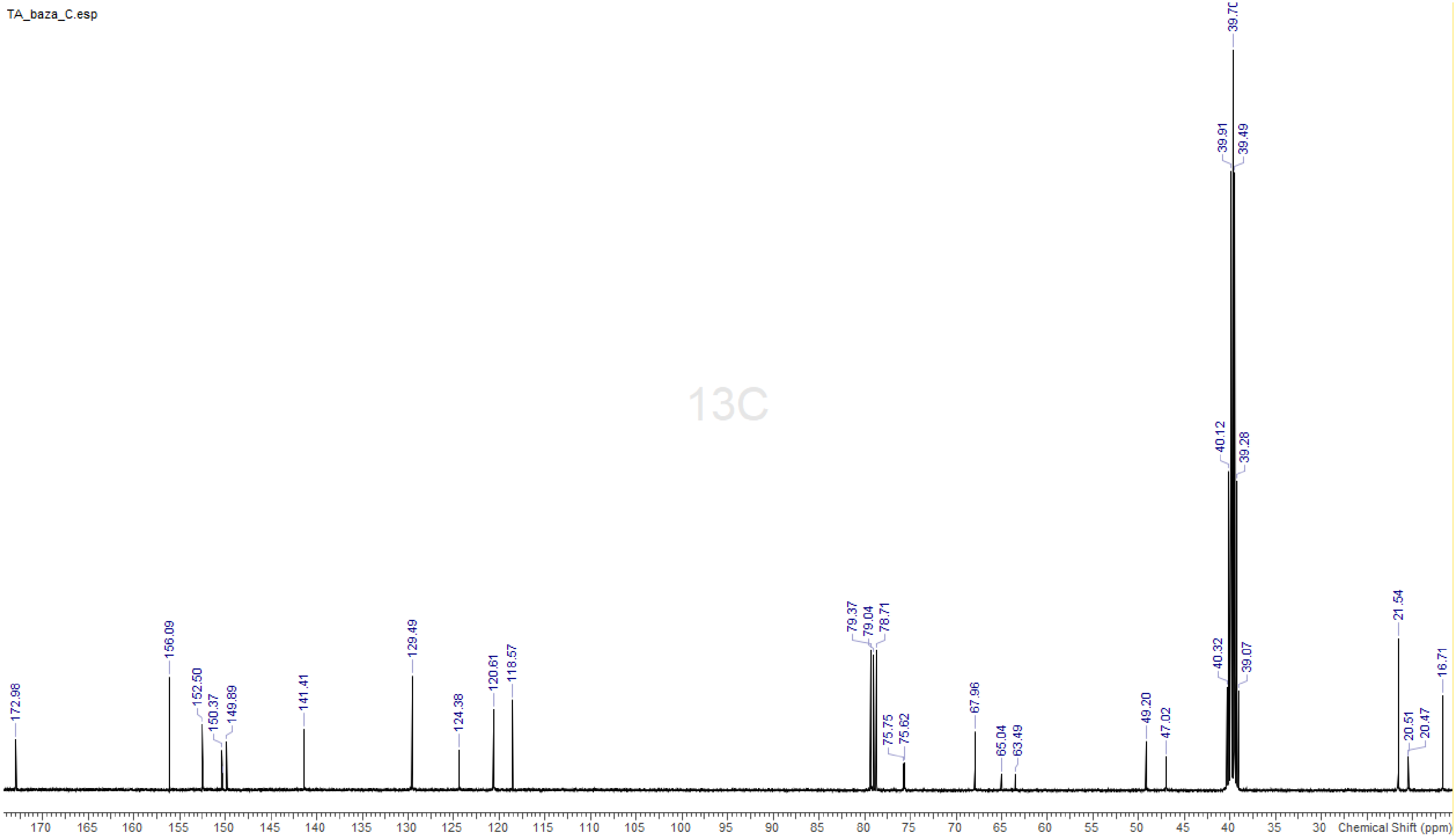

Figure S13. ^{13}C-NMR spectrum of tenofovir alafenamide (TA).

3.2. Tenofovir alafenamide hydrochloride (TA HCl)

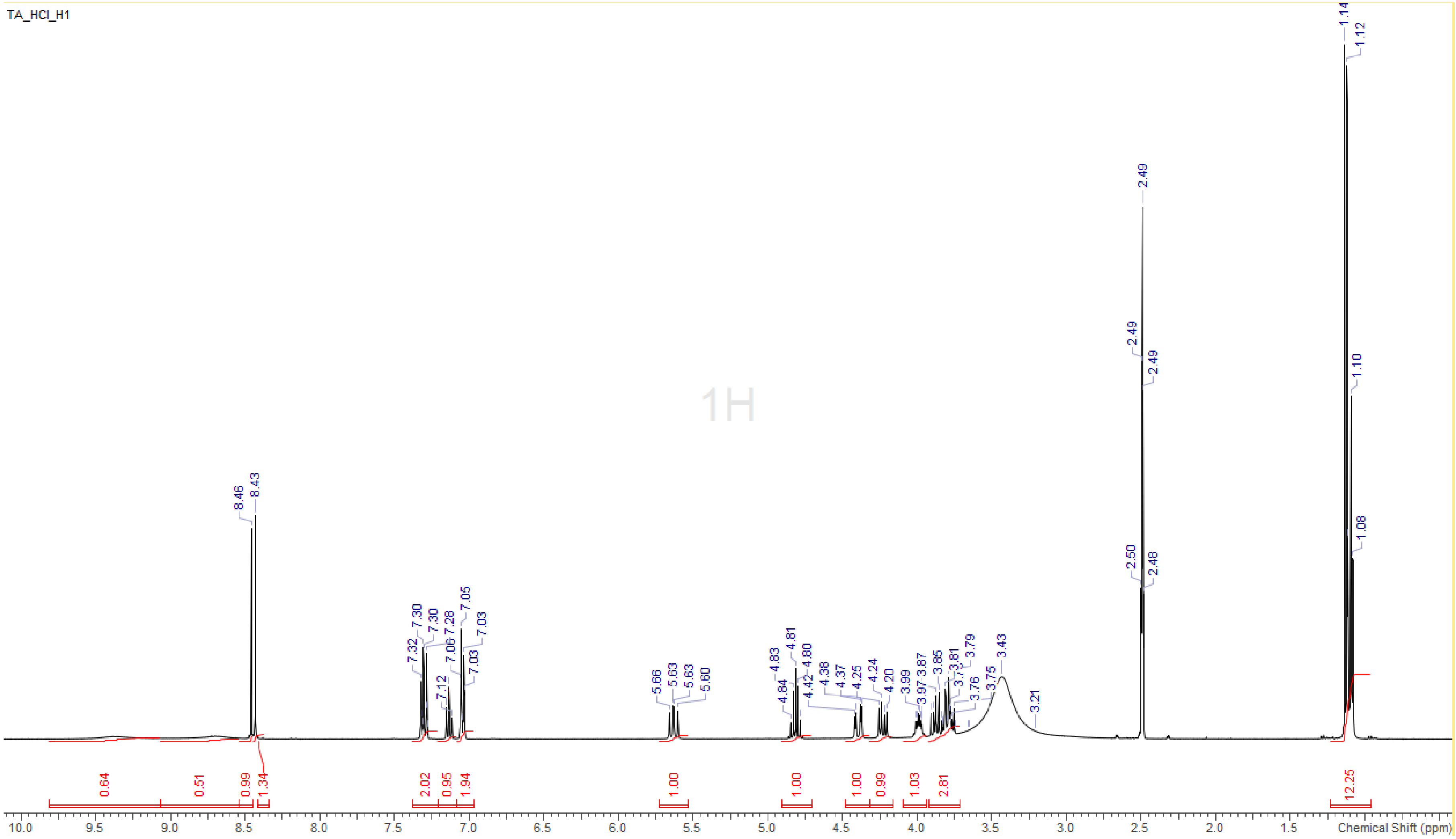

Figure S14.^{1}H-NMR spectrum of tenofovir alafenamide hydrochloride (TA HCl).

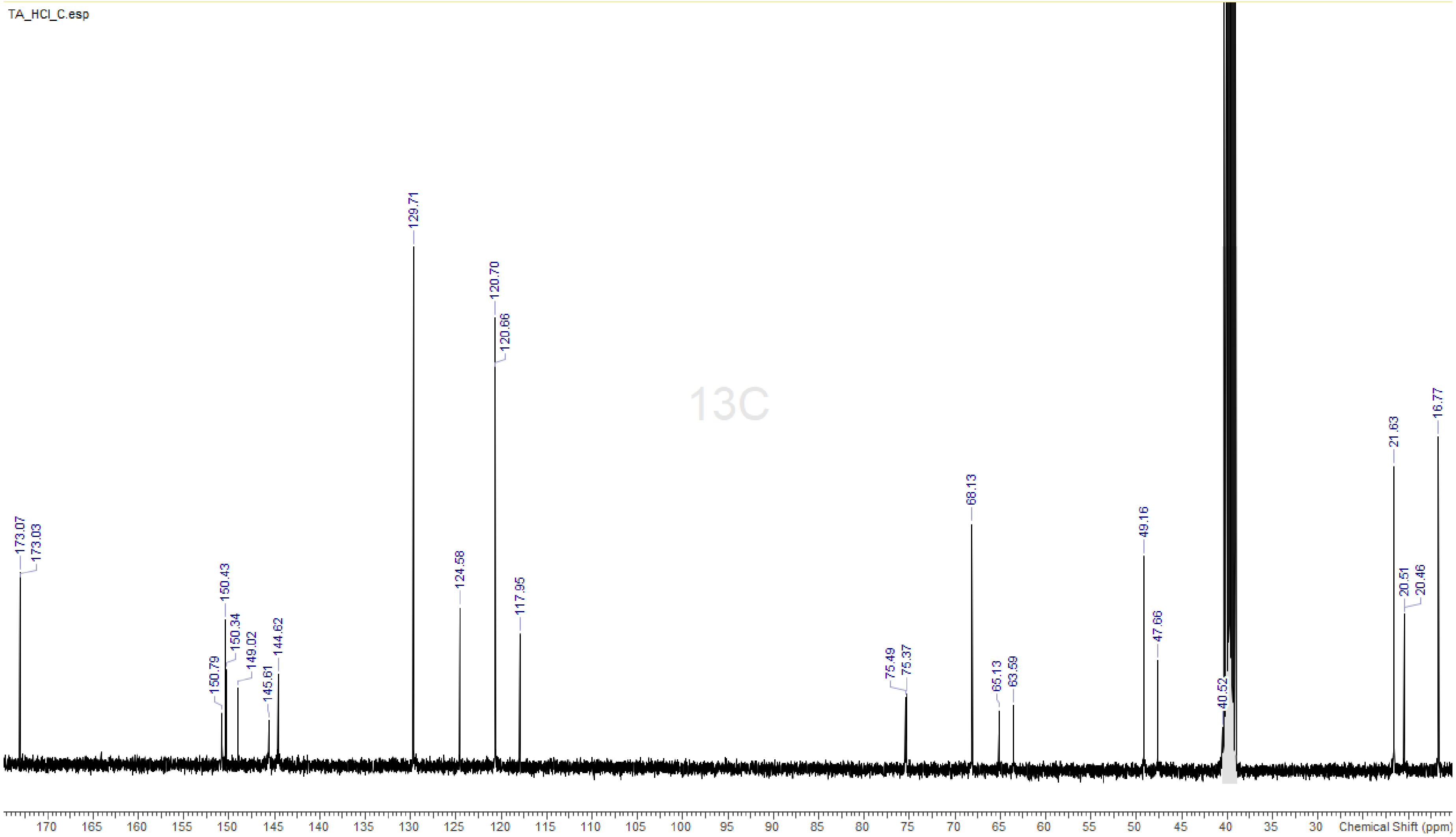

Figure S15. ^{13}C-NMR spectrum of tenofovir alafenamide hydrochloride (TA HCl).

3.3. Tenofovir alafenamide hemifumarate (TA HF)

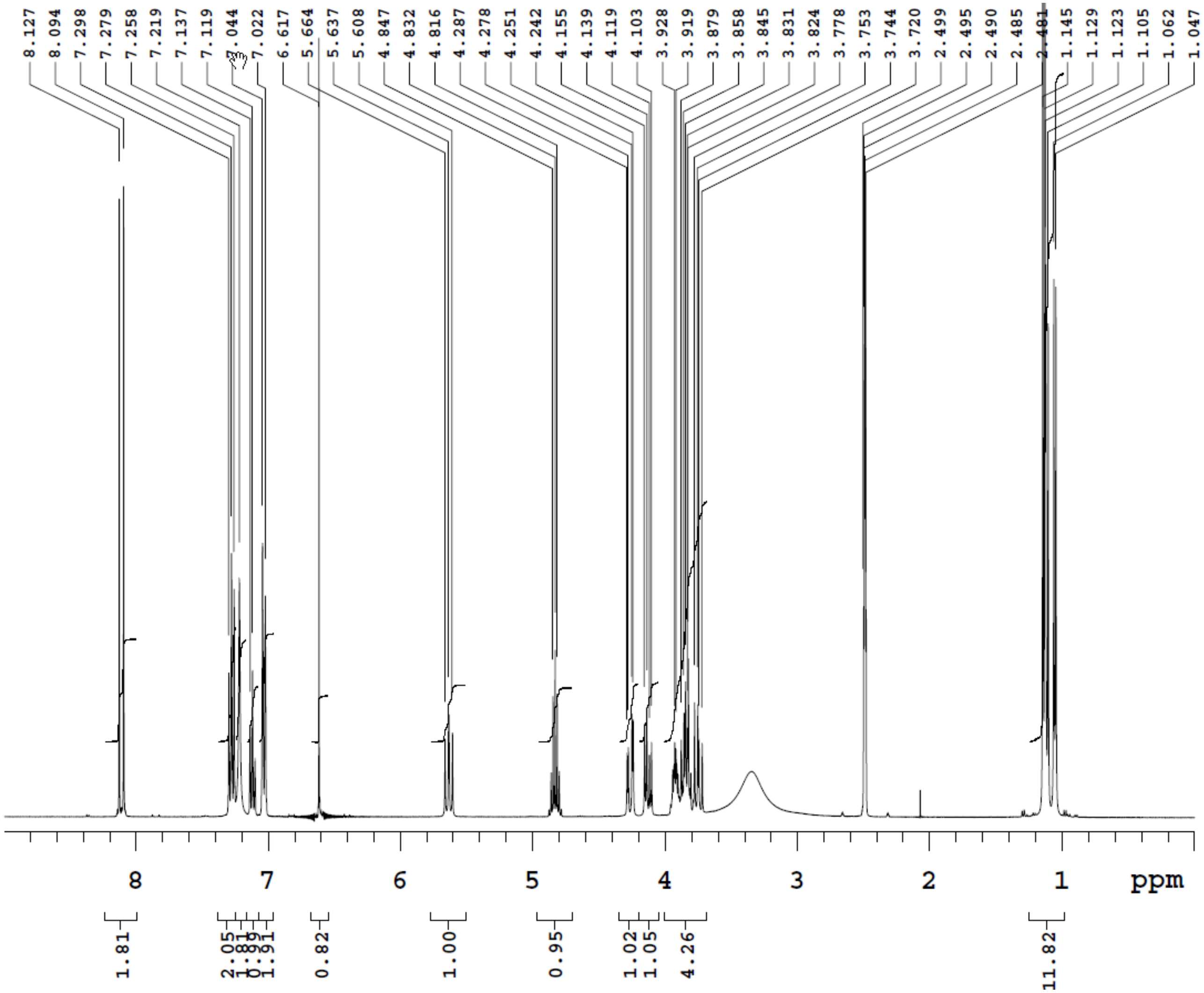

Figure S16.^{1}H-NMR spectrum of tenofovir alafenamide hemifumarate (TA HF).

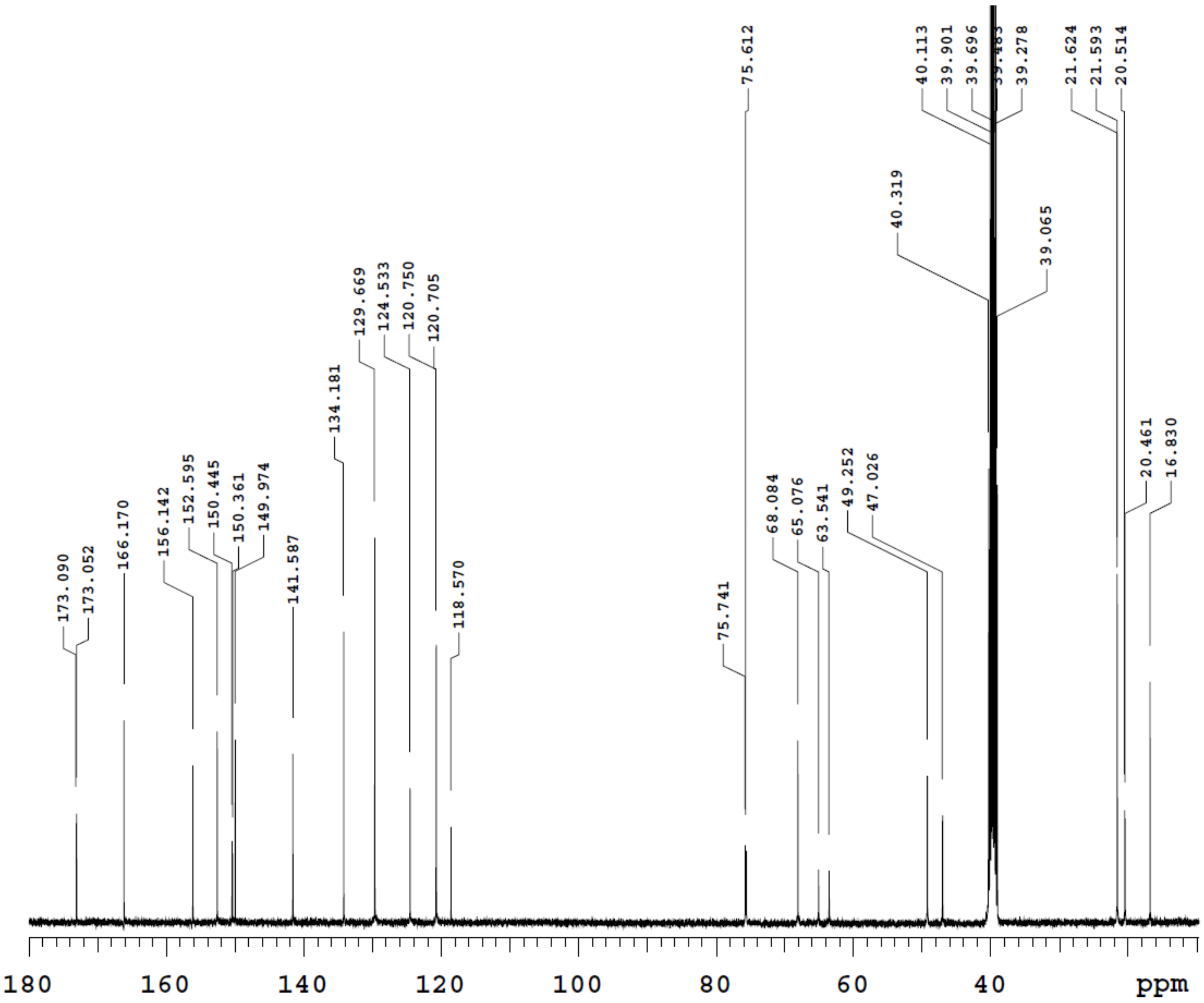

Figure S17. ^{13}C-NMR spectrum of tenofovir alafenamide hemifumarate (TA HF).

3.4. Tenofovir alafenamide monofumarate form I (TA MF1)

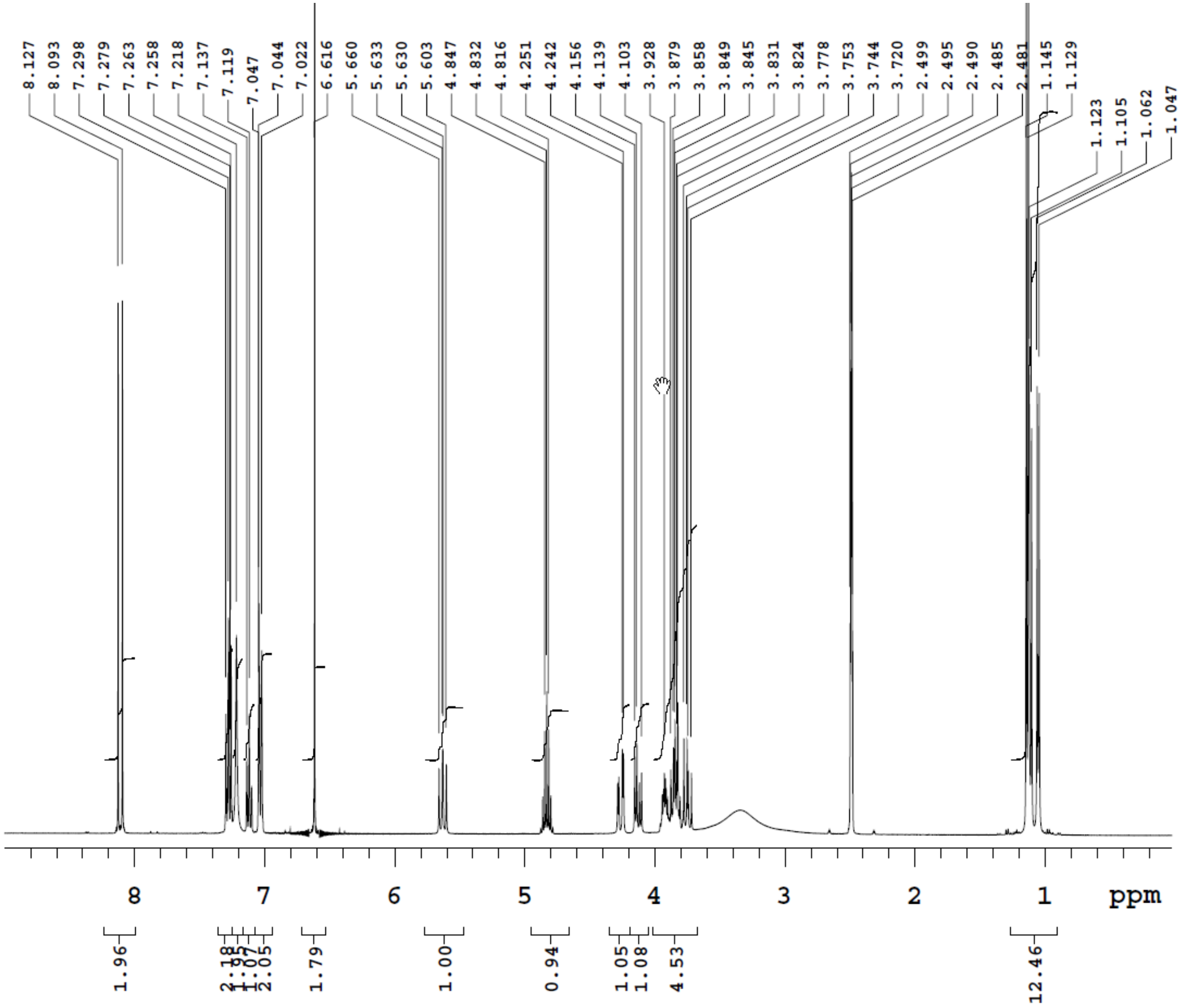

Figure S18.^{1}H-NMR spectrum of tenofovir alafenamide monofumarate form I (TA MF1).

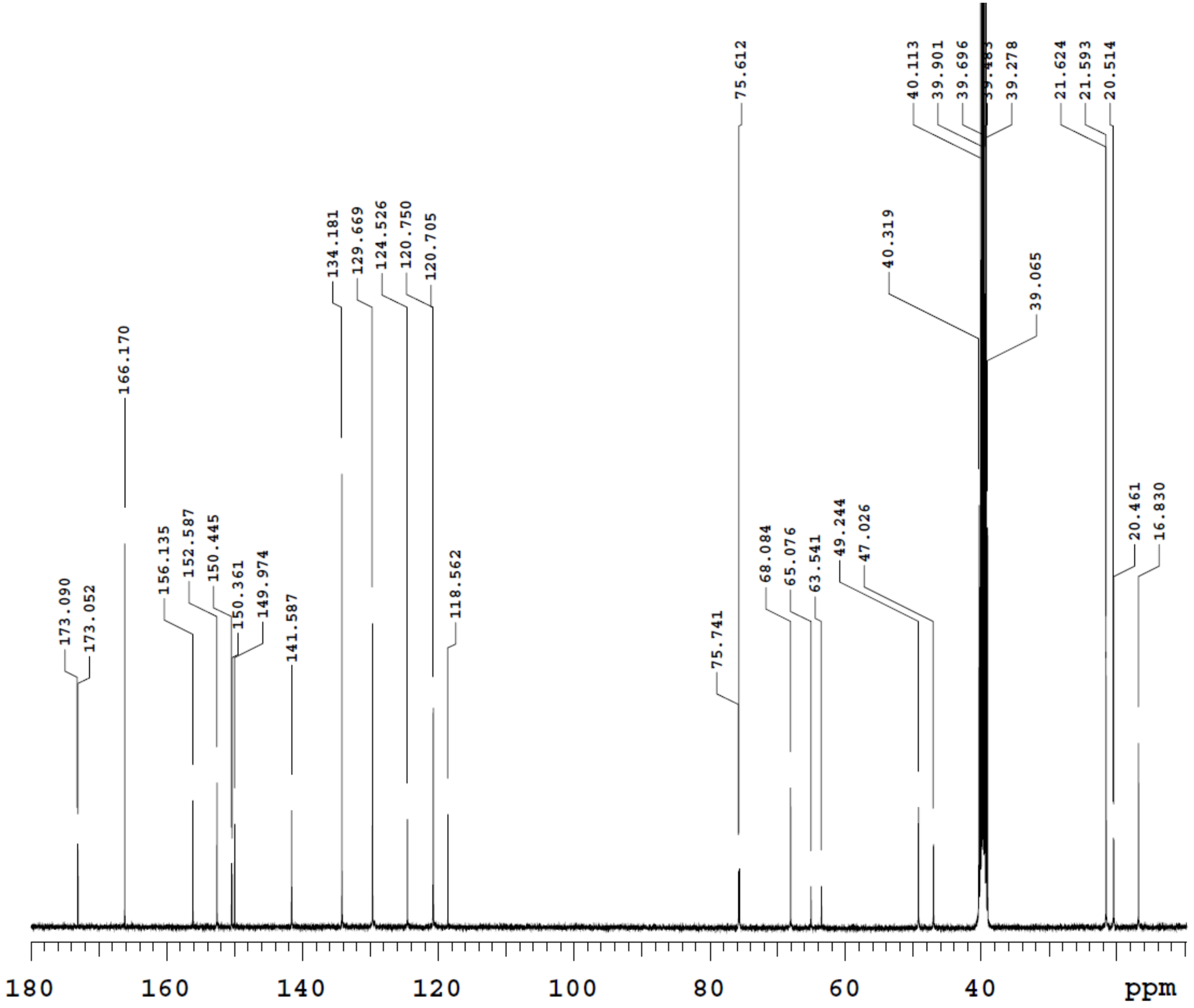

Figure S19. ^{13}C-NMR spectrum of tenofovir alafenamide monofumarate form I (TA MF1).

3.5. Tenofovir alafenamide monofumarate form II (TA MF2)

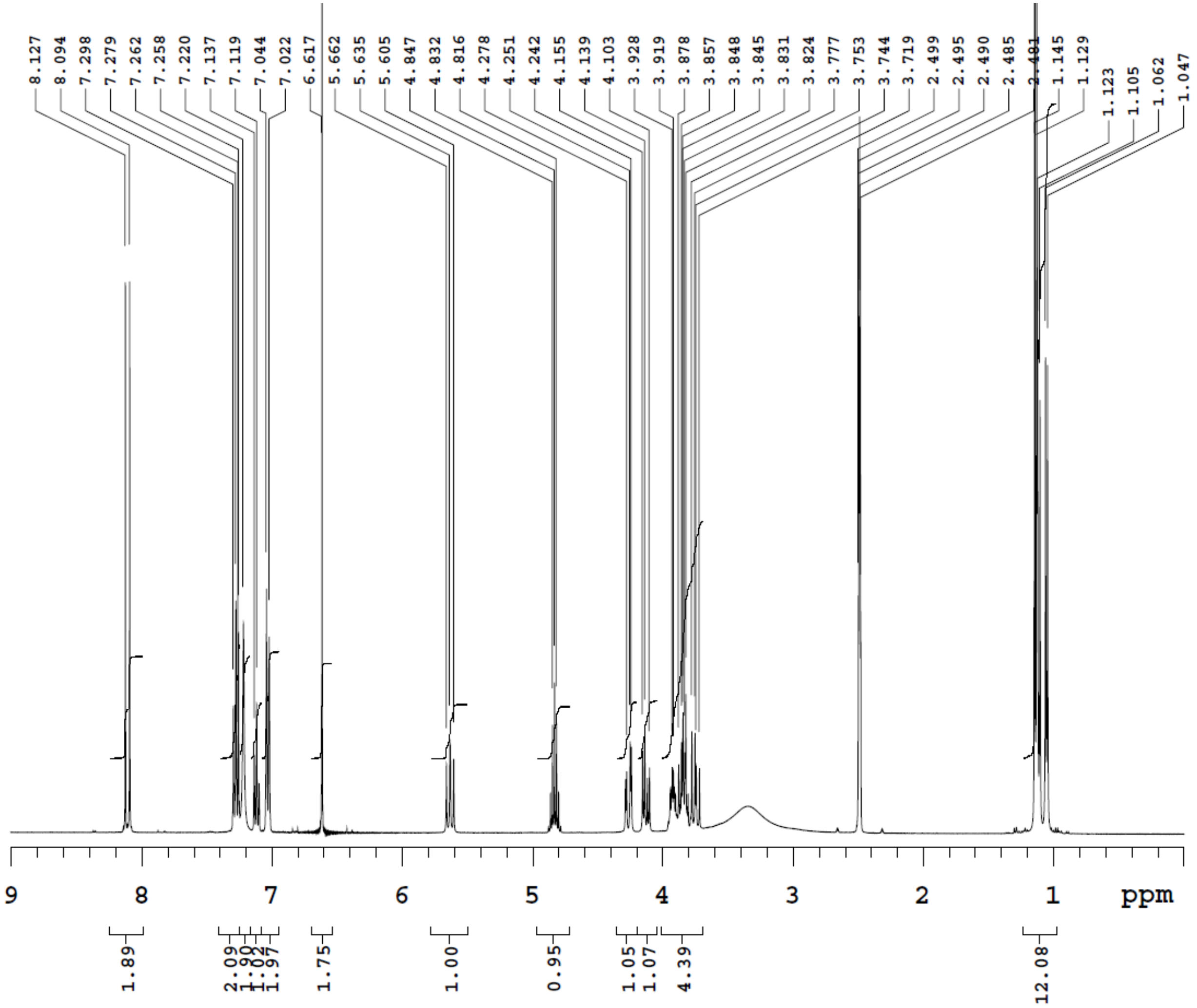

Figure S20.^{1}H-NMR spectrum of tenofovir alafenamide monofumarate form II (TA MF2).

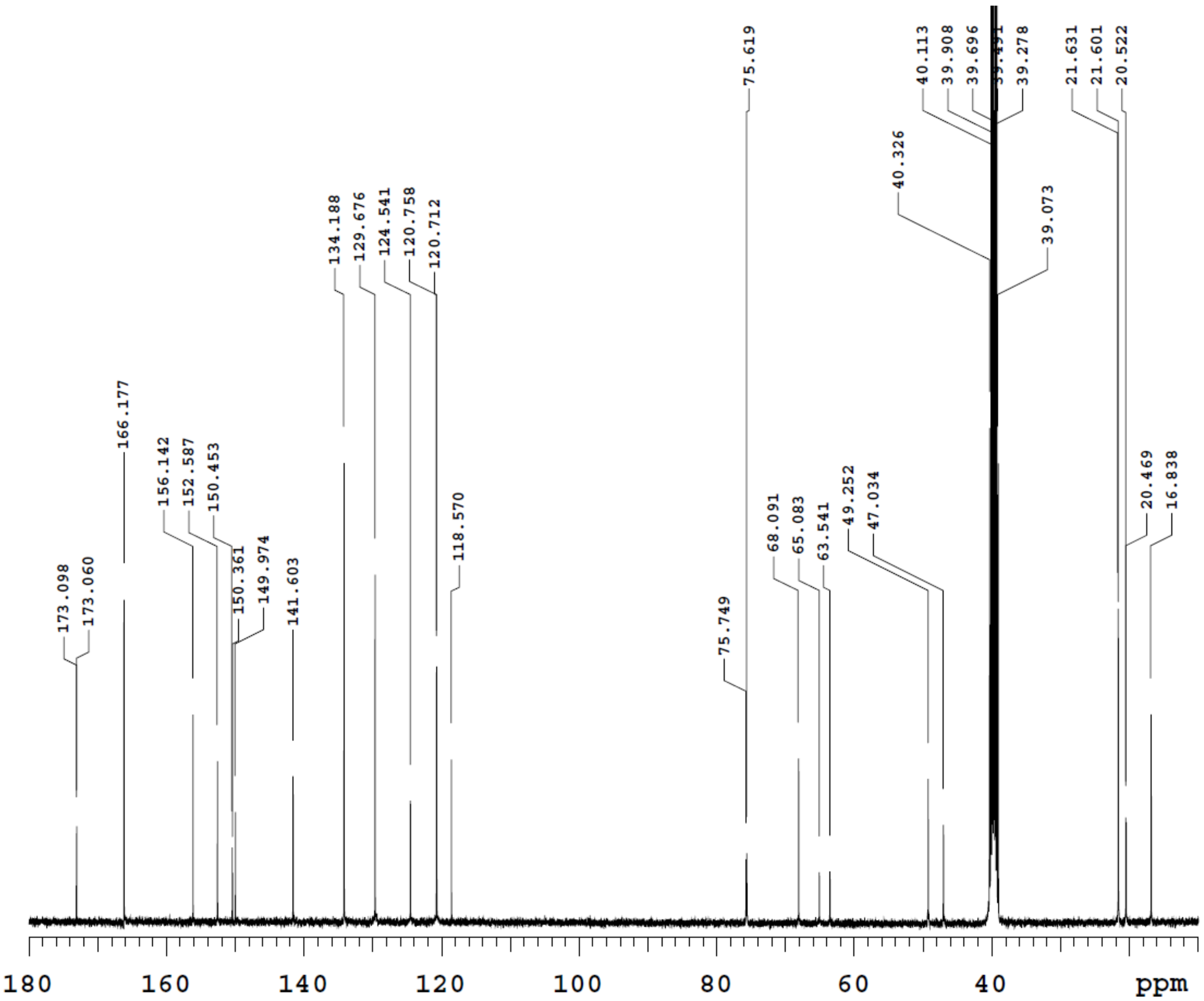

Figure S21. ^{13}C-NMR spectrum of tenofovir alafenamide monofumarate form II (TA MF2).

3.6. Tenofovir alafenamide monofumarate form III (TA MF3)

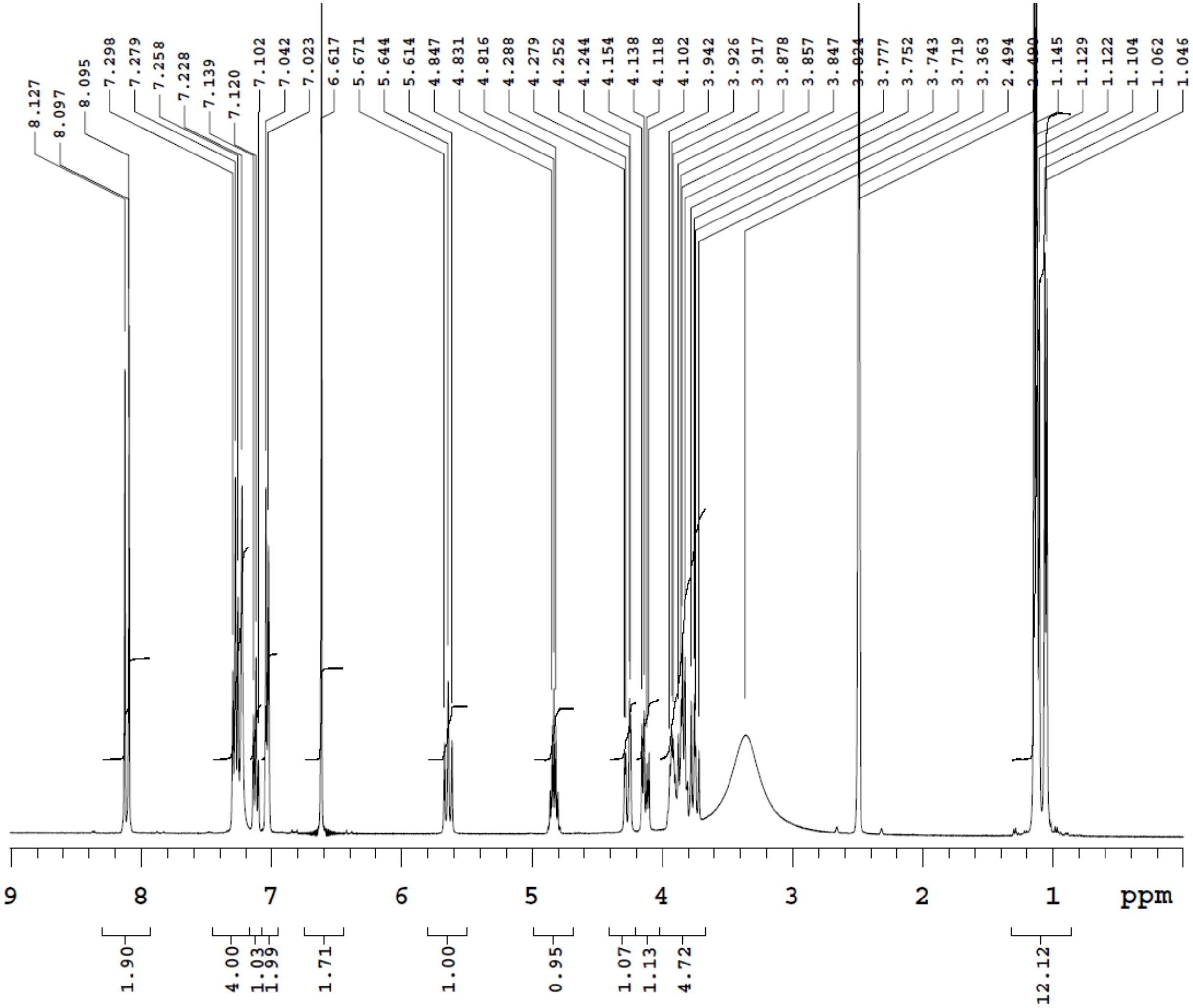

Figure S22. ^{1}H-NMR spectrum of tenofovir alafenamide monofumarate form III (TA MF3).

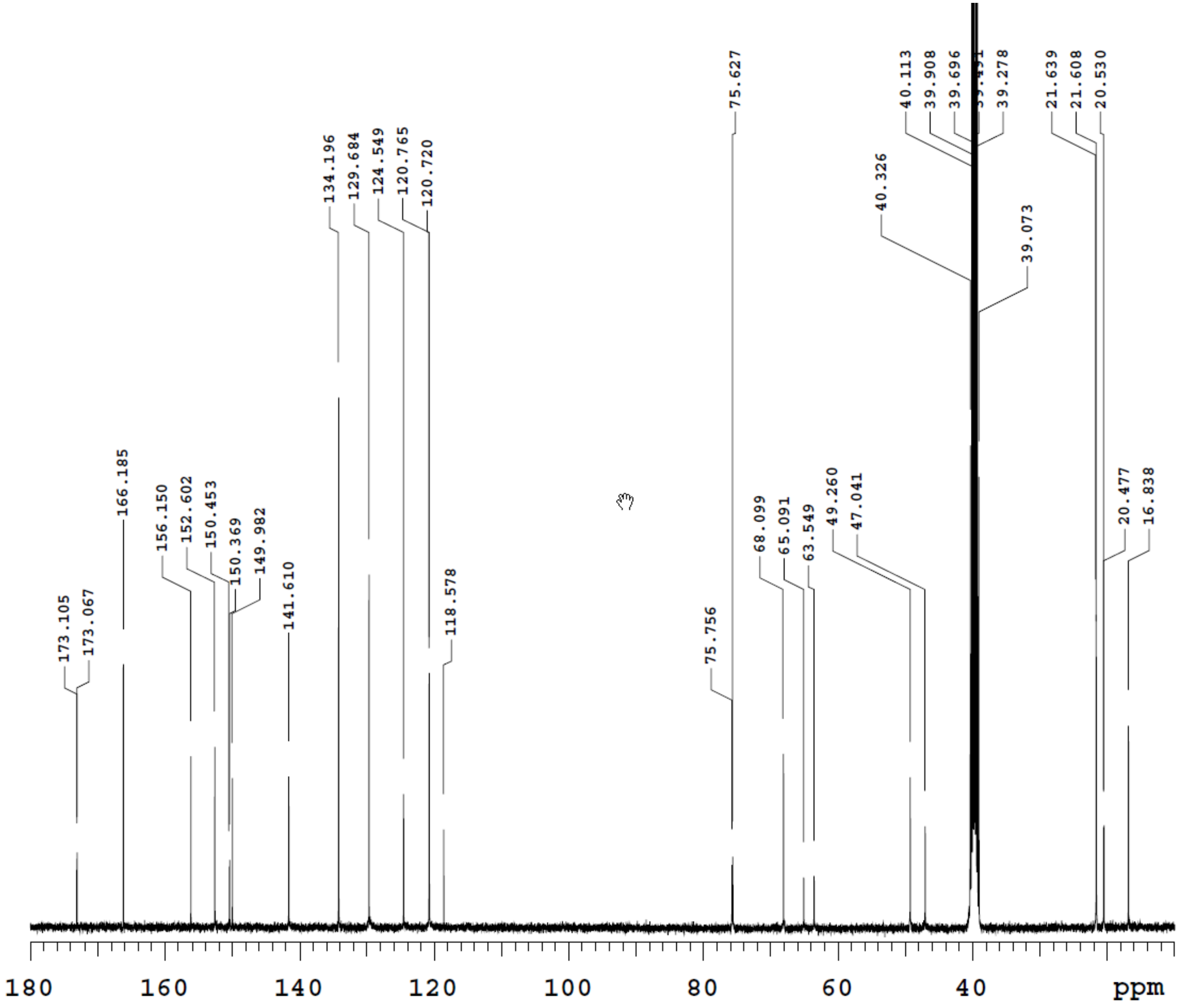

Figure S23. ^{13}C-NMR spectrum of tenofovir alafenamide monofumarate form III (TA MF3).

4. ssNMR spectra of tenofovir alafenamide derivatives

4.1. [1]*H echo MAS NMR spectra*

Tenofovir alafenamide (TA)

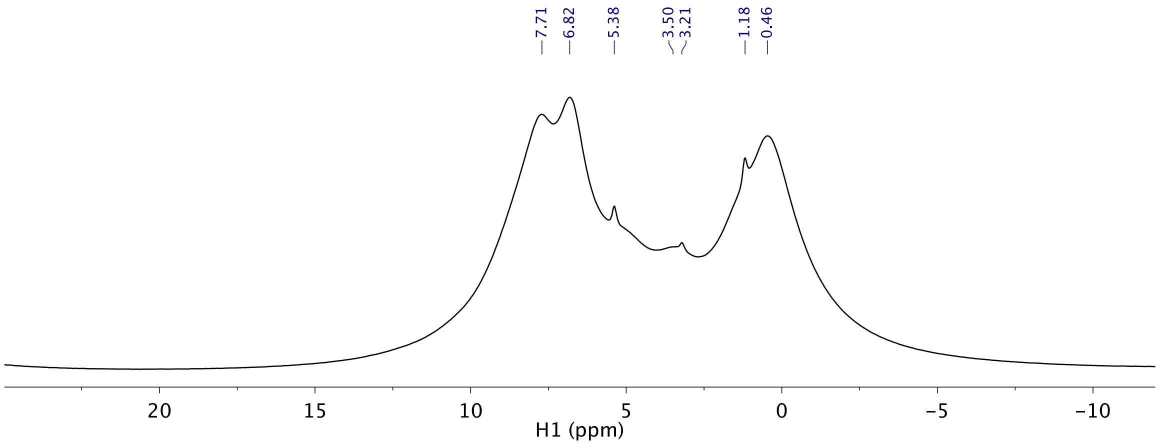

Tenofovir alafenamide hydrochloride salt (TA HCl)

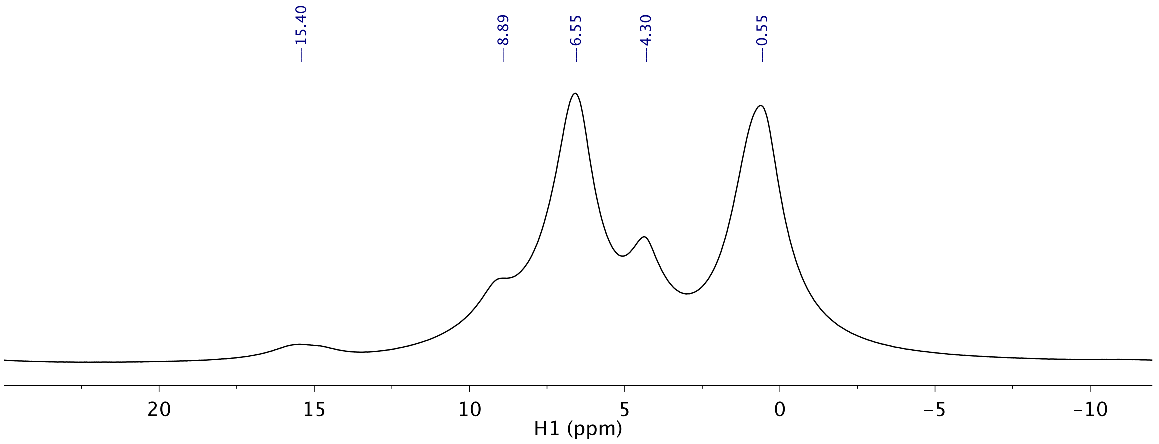

Tenofovir alafenamide hemifumarate (TA HF)

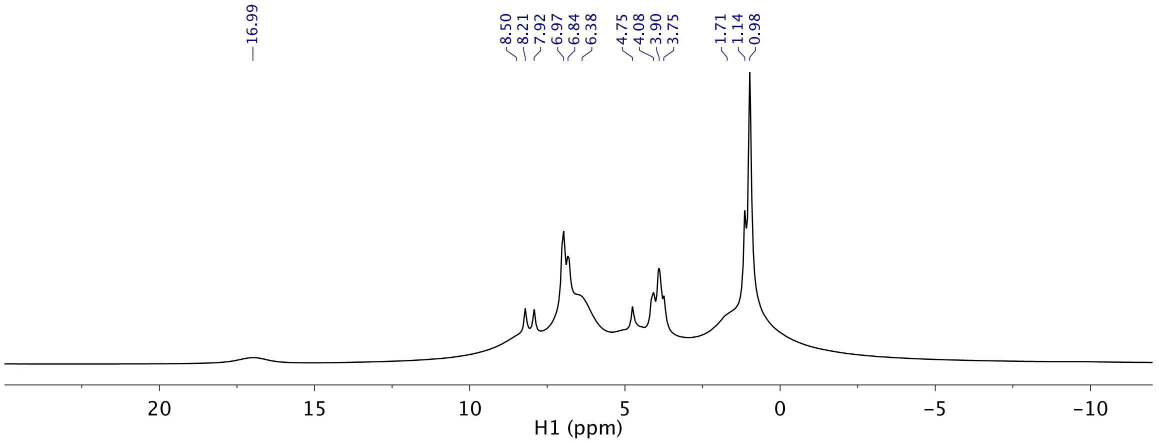

Figure S24.^{1}H echo MAS NMR spectra of tenofovir alafenamide (TA), tenofovir alafenamide hydrochloride salt (TA HCl) and tenofovir alafenamide hemifumarate (TA HF).

Tenofovir alafenamide monofumarate I (TA MF1)

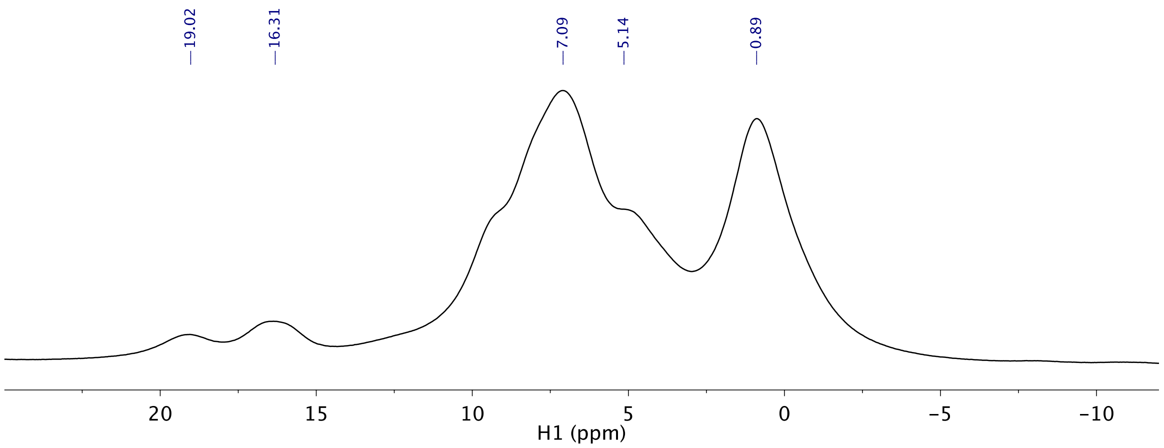

Tenofovir alafenamide monofumarate II (TA MF2)

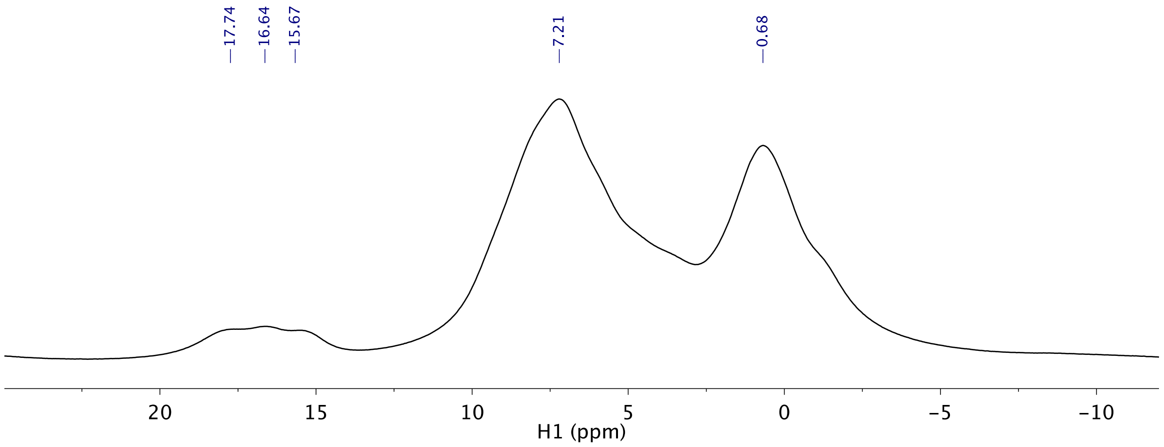

Tenofovir alafenamide monofumarate III (TA MF3)

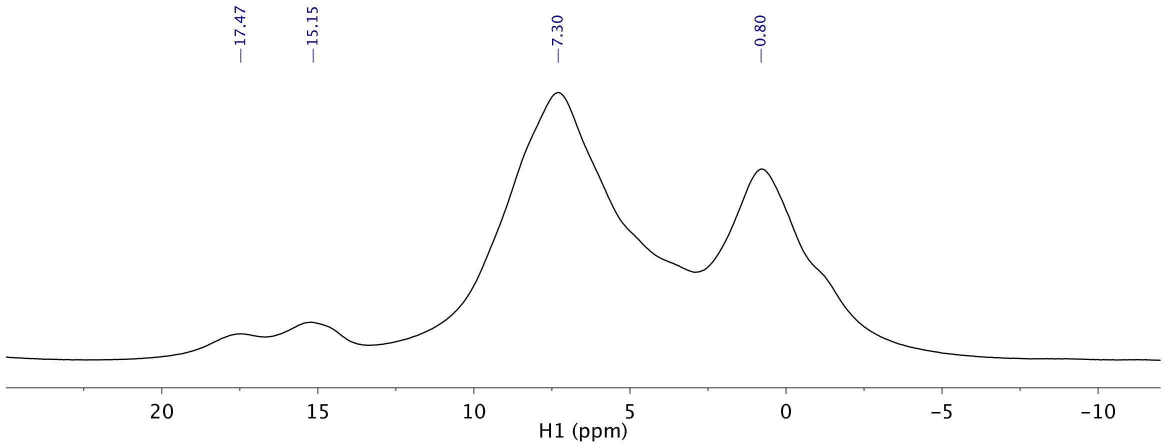

Figure S25. ^{1}H echo MAS NMR spectra of tenofovir alafenamide monofumarate I (TA MF1), tenofovir alafenamide monofumarate II (TA MF2) and tenofovir alafenamide monofumarate III (TA MF3).

4.2. ^{15}N CP MAS NMR spectra of tenofovir alafenamide monofumarates

Tenofovir alafenamide monofumarate I (TA MF1)

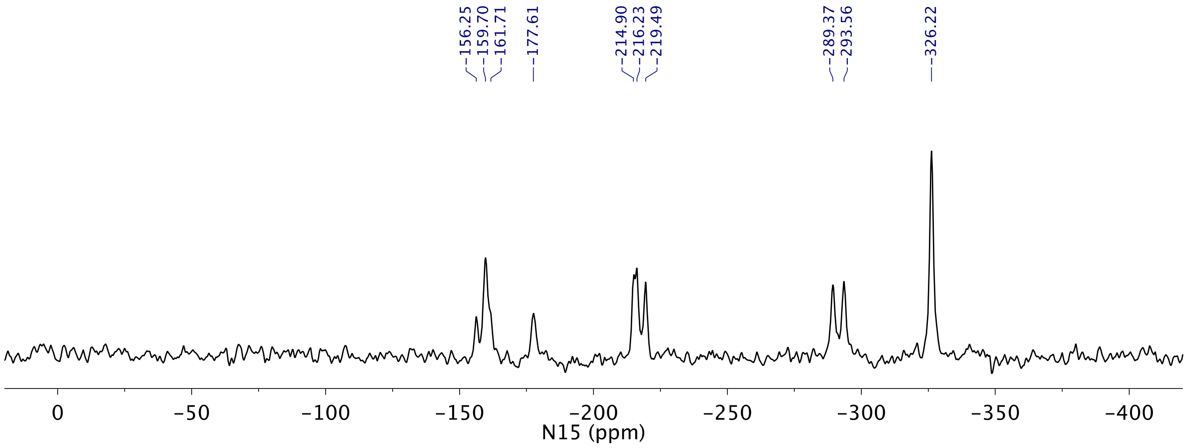

Tenofovir alafenamide monofumarate II (TA MF2)

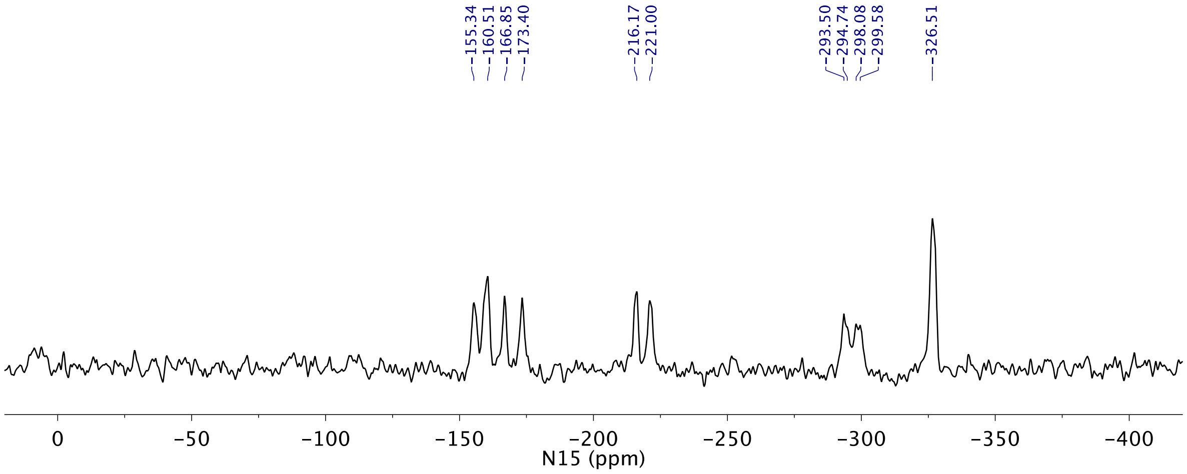

Tenofovir alafenamide monofumarate III (TA MF3)

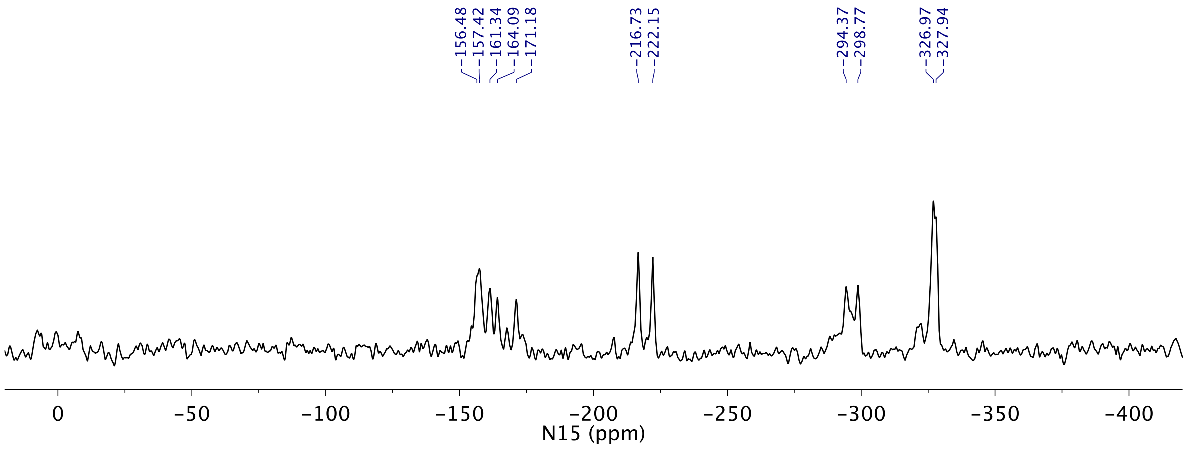

Figure S26. ^{15}N CP MAS NMR spectra of tenofovir alafenamide monofumarate I (TA MF1), tenofovir alafenamide monofumarate II (TA MF2) and tenofovir alafenamide monofumarate III (TA MF3).

4.3. ^{15}N CP MAS NMR spectra of tenofovir alafenamide monofumarates

Tenofovir alafenamide monofumarate I (TA MF1)

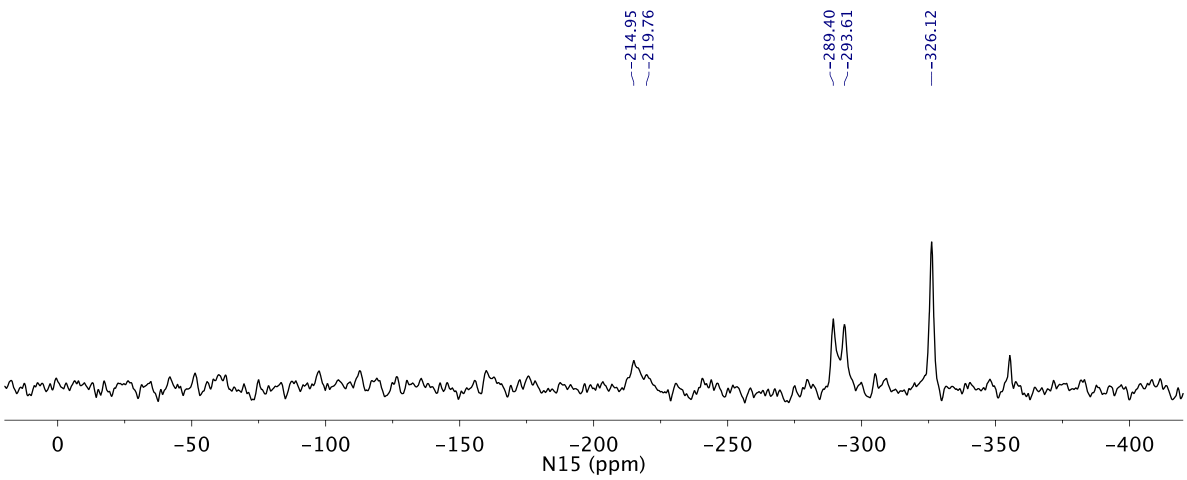

Tenofovir alafenamide monofumarate II (TA MF2)

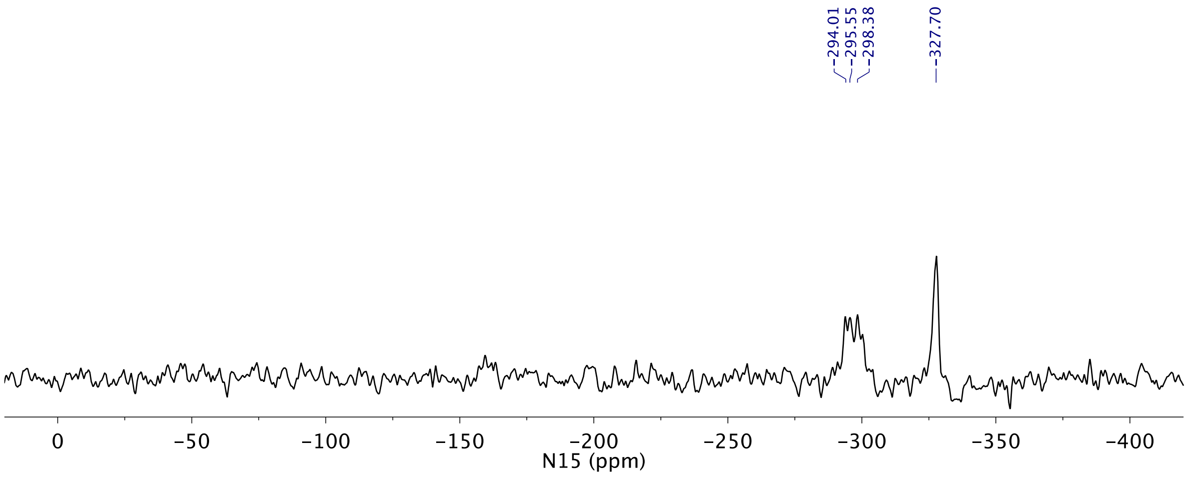

Tenofovir alafenamide monofumarate III **(TA MF3)**

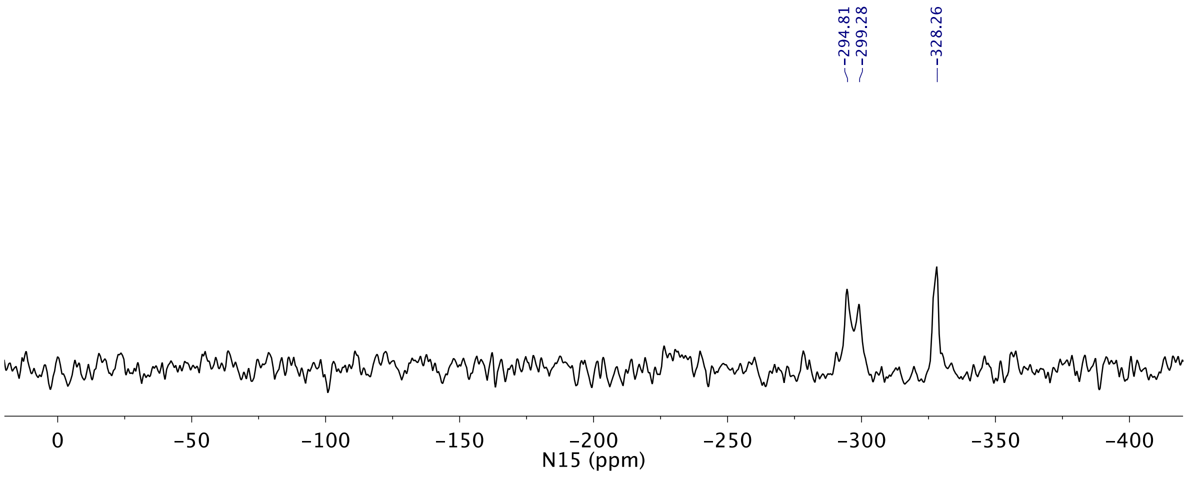

Figure S27. ^{15}N LG-CP MAS NMR spectra of tenofovir alafenamide monofumarate I (TA MF1), tenofovir alafenamide monofumarate II (TA MF2) and tenofovir alafenamide monofumarate III (TA MF3). Experimental conditions were kept the same as for reference substances TA free base and TA HCl. Later, S/N ratio was improved for ^{15}N LG-CP MAS NMR spectra of tenofovir alafenamide monofumarates and the spectra are shown in Figure 8.

5. Tables of Data

5.1. p-XRD data for TA HF, TA MF1, TA MF2 and TA MF3

Table S1. *p*-XRD Data for TA HF, TA MF1, TA MF2 and TA MF3.

TA HF		TA MF1		TA MF2		TA MF3	
Pos. [°2θ]	Rel. Int. [%]	Pos. [°2θ]	Rel. Int. [%]	Pos. [°2θ]	Rel. Int. [%]	Pos. [°2θ]	Rel. Int. [%]
6.9	100	5.3	80	5.6	16	5.4	11
8.5	87	9.8	6	7.3	12	5.6	10
9.7	6	10.4	100	9.4	45	7.3	4
10.0	9	11.0	68	10.1	100	9.4	35
11.0	81	11.3	7	10.9	35	9.8	43
11.1	5	11.6	5	11.4	55	10.2	19
12.0	16	12.3	18	12.2	6	10.6	100
12.2	21	13.4	2	13.0	18	11.2	11
13.8	7	13.8	4	14.0	17	11.6	24
14.0	3	14.4	12	14.4	34	12.2	6
14.7	4	14.9	7	14.7	9	12.6	17
14.8	7	15.6	9	15.1	6	13.3	41
15.5	3	15.9	9	15.5	5	14.2	54
15.8	35	16.2	7	16.9	9	14.6	12
16.2	36	16.6	7	17.5	36	15.1	5
16.6	9	17.3	2	17.8	20	16.4	5
17.1	7	17.7	22	18.6	9	17.0	69
17.6	20	18.7	24	18.8	11	17.2	12
18.0	13	19.0	25	19.2	41	17.7	59
18.3	19	19.5	48	19.6	34	18.1	4
18.6	12	20.6	9	20.5	19	18.8	26
19.6	3	20.8	13	21.0	64	19.2	33
20.2	26	21.2	29	21.3	37	19.6	41
20.8	44	21.9	15	21.5	35	20.2	8
21.1	3	22.3	10	21.8	35	20.6	12
21.4	11	22.6	12	22.7	98	20.9	34
21.6	13	22.9	9	23.9	15	21.4	46
22.0	2	23.6	18	25.4	12	21.6	51
22.5	7	23.9	4	26.0	7	21.9	17
23.0	8	24.8	7	26.6	8	22.6	60
23.1	12	26.6	38	28.4	40	23.7	24
23.3	16	27.1	17			24.5	12
24.1	6	27.8	7			24.9	8
24.5	6	28.1	4			25.3	10
24.8	14	28.9	3			25.9	8
25.0	12	29.5	2			26.2	6
25.3	4					26.7	5
25.5	6					27.7	8
25.8	5					28.3	22
26.5	11					28.7	8
27.0	4					29.6	7
27.6	3						
27.8	9						
28.3	2						
28.7	6						
29.1	2						
29.6	3						

© 2020 by the authors. Submitted for possible open access publication under the terms and conditions of the Creative Commons Attribution (CC BY) license (http://creativecommons.org/licenses/by/4.0/).
